# Preclinical *ex-vivo* Testing of Anti-inflammatory Drugs in a Bovine Intervertebral Degenerative Disc Model

**DOI:** 10.3389/fbioe.2020.00583

**Published:** 2020-06-10

**Authors:** Zhen Li, Yannik Gehlen, Fabian Heizmann, Sibylle Grad, Mauro Alini, R. Geoff Richards, David Kubosch, Norbert Südkamp, Kaywan Izadpanah, Eva Johanna Kubosch, Gernot Lang

**Affiliations:** ^1^AO Research Institute Davos, Davos, Switzerland; ^2^Department of Orthopedics and Trauma Surgery, Faculty of Medicine, Medical Center - Albert-Ludwigs-University of Freiburg, Albert-Ludwigs-University of Freiburg, Freiburg, Germany

**Keywords:** spine, regeneration, inflammation, intervertebral disc, disc degeneration, organ culture, bioreactor, 3R

## Abstract

Discogenic low back pain (LBP) is a main cause of disability and inflammation is presumed to be a major driver of symptomatic intervertebral disc degeneration (IDD). Anti-inflammatory agents are currently under investigation as they demonstrated to alleviate symptoms in patients having IDD. However, their underlying anti-inflammatory and regenerative activity is poorly explored. The present study sought to investigate the potential of Etanercept and Tofacitinib for maintaining disc homeostasis in a preclinical intervertebral disc (IVD) organ culture model within IVD bioreactors allowing for dynamic loading and nutrient exchange. Bovine caudal IVDs were cultured in a bioreactor system for 4 days to simulate physiological or degenerative conditions: (1) Phy—physiological loading (0.02–0.2 MPa; 0.2 Hz; 2 h/day) and high glucose DMEM medium (4.5 g/L); (2) Deg+Tumor necrosis factor α (TNF-α)—degenerative loading (0.32–0.5 MPa; 5 Hz; 2 h/day) and low glucose DMEM medium (2 g/L), with TNF-α injection. Etanercept was injected intradiscally while Tofacitinib was supplemented into the culture medium. Gene expression in the IVD tissue was measured by RT-qPCR. Release of nitric oxide (NO), interleukin 8 (IL-8) and glycosaminoglycan (GAG) into the IVD conditioned medium were analyzed. Cell viability in the IVD was assessed using lactate dehydrogenase and ethidium homodimer-1 staining. Immunohistochemistry was performed to assess protein expression of IL-1β, IL-6, IL-8, and collagen type II in the IVD tissue. Etanercept and Tofacitinib downregulated the expression of IL-1β, IL-6, IL-8, Matrix metalloproteinase 1 (MMP1), and MMP3 in the nucleus pulposus (NP) tissue and IL-1β, MMP3, Cyclooxygenase-2 (COX2), and Nerve growth factor (NGF) in the annulus fibrosus (AF) tissue. Furthermore, Etanercept significantly reduced the IL-1β positively stained cells in the outer AF and NP regions. Tofacitinib significantly reduced IL-1β and IL-8 positively stained cells in the inner AF region. Both, Etanercept and Tofacitinib reduced the GAG loss to the level under physiological culture condition. Etanercept and Tofacitinib are able to neutralize the proinflammatory and catabolic environment in the IDD organ culture model. However, combined anti-inflammatory and anabolic treatment may be required to constrain accelerated IDD and relieving inflammation-induced back pain.

## Introduction

Low back pain (LBP) is a main cause for disability and early retirement and remains an immense socioeconomic burden in modern societies (Hoy et al., 2014; Buchbinder et al., 2018; Clark and Horton, 2018). Symptomatic intervertebral disc degeneration (IDD) is one of the major causes of LBP and is characterized by early degradation of extracellular matrix (ECM), release of proinflammatory cytokines and altered spine biomechanics (Pye et al., 2004; Le Maitre et al., 2007a; Podichetty, 2007; Freemont, 2009). The pathophysiology of discogenic pain is complex and involves various factors such as trauma, mechanical overloading, oxidative stress, metabolic disorders, genetic preposition, and inflammation interacting with the peripheral and the central nervous system (Stirling et al., 2001; Rannou et al., 2003; Battie et al., 2019; Fujii et al., 2019). It is hypothesized, that IDD is associated with a “first hit” causing structural damage to the intervertebral disc (IVD) followed by an inflammatory response within the microenvironment of the IVD (Risbud and Shapiro, 2014). Proinflammatory cytokines such as tumor necrosis factor alpha (TNF-α) and interleukin 1 beta (IL-1β) are considered of crucial importance in the pathogenesis of IDD as they link the inflammatory process to accelerated tissue degeneration and pain (Risbud and Shapiro, 2014). Recent studies indicated that the presence of TNF-α causes an upregulation of major catabolic enzymes involving Matrix-Metalloproteinases (MMPs) and A Disintegrin and Metalloprotease with Thrombospondin Motifs (ADAMTS), contributing to advanced structural decay in IDD (Purmessur et al., 2013; Tian et al., 2013; Baptista et al., 2020; Zhou et al., 2020). Moreover, TNF-α is associated with the development of discogenic pain by upregulating pain transmitter substance P (SP) and by attracting nerve ingrowth into the outer annulus fibrosus (Freemont et al., 1997, 2002; Olmarker and Larsson, 1998; Igarashi et al., 2000; Risbud and Shapiro, 2014; Evashwick-Rogler et al., 2018).

Current therapies for discogenic pain include conservative as well as surgical treatment in severe scenarios, such as discectomy and fusion. Although commonly performed, the true benefit of current surgical therapies in patients with discogenic LBP remains questionable (Lurie et al., 2014). Presently, there are no causative therapeutic options for patients with failed conservative treatment who do not qualify for surgery.

In order to overcome current treatment barriers and improve patient care, biological approaches on IVD regeneration have gained increasing interest. According to the state of IVD degeneration, staged therapeutic concepts have been developed such as biomolecular, cell therapy, as well as tissue engineering (TE)-based treatments (Li et al., 2014, 2015, 2017; Pirvu et al., 2015; Mojica-Santiago et al., 2018; Pennicooke et al., 2018). In earlier stages of disc degeneration when viable cells are still present, the aim is to relieve pain and enhance the intradiscal metabolism by stopping the catabolic cascade of ECM breakdown and inflammation. As inflammation is presumed to be a major driver of symptomatic IDD, the use of anti-inflammatory biologicals as a novel treatment strategy for early stage IDD is a promising approach to consider.

TNF-α inhibitors, such as Infliximab, Adalimumab, or Etanercept, have been investigated for the treatment of rheumatoid arthritis (RA) and inflammatory bowel disease, and are commercially available for more than 15 years. These drugs are currently under investigation for the treatment of IDD, as observational studies demonstrated to partially alleviate discogenic as well as sciatic leg pain and reduce the risk for spine surgery when local application is performed (Cooper and Freemont, 2004; Genevay et al., 2004, 2012; Korhonen et al., 2004, 2006; Tobinick and Davoodifar, 2004; Autio et al., 2006; Cohen et al., 2009; Okoro et al., 2010; Ohtori et al., 2012; Williams et al., 2013; Pimentel et al., 2014; Sainoh et al., 2016). However, the encouraging findings of observational reports could not be confirmed in randomized controlled trials and recent meta-analysis failed to demonstrate the superiority of anti-TNF-a therapies. Nevertheless, studies indicated that Anti-TNF-a therapies reduced the risk of having spine surgery in cases of sciatica. All authors of the meta-analysis agreed that larger and better-designed studies may need to be performed on anti-TNF-a therapies against placebo since all included studies comprised small study populations. Although evidence exists to reduce symptoms, the detailed biological and mechanical effects of TNF-α inhibitors on degenerated IVDs need to be investigated before broad application can be implemented in patients with refractory discogenic pain.

In 2017, a novel orally administered anti-inflammatory agent, Tofacitinib, was licensed for the treatment of refractory RA in Europe (Milici et al., 2008; van Vollenhoven et al., 2012). Tofacitinib irreversibly inhibits Janus Kinase (JAK) 1 and 3, reduces symptoms and improves physical function in patients with RA (Fleischmann et al., 2012). TNF-α was shown to activate the JAK/signal transducers and activators of transcription (STAT) pathway, which serves as a decisive pathway for many proinflammatory cytokines, such as interleukin 6 (IL-6). The JAK-STAT3 participates in the pathogenesis of IDD and selective JAK-inhibition suppressed degenerative effects of pro-inflammatory cytokines in rat annulus fibrosus (AF) cells in a recent *in vitro* study (Suzuki et al., 2017). It is unknown whether the JAK-inhibitor Tofacitinib would show similar effects under more relevant IDD *ex vivo* experimental conditions compared to cell culture studies. Additionally, the cytocompatibility of these drugs toward disc cells needs to be determined if local administration is desired.

*In vitro* cell culture studies fail to simulate the complex microenvironment within IVDs realistically as they lack to represent the harsh and avascular 3D niche and/or dynamic loading conditions of the IVD. Recently, we have established a bovine degenerative *ex vivo* organ culture model to mimic the proinflammatory and mechanical micro-environment within an early stage degenerative IVD (Lang et al., 2018). This model is highly valuable as it allows for rapid and cost-efficient screening of novel drug therapies under relevant conditions.

The purpose of the present study was to evaluate the anti-inflammatory and anti-catabolic potential of TNF-α inhibitor Etanercept and the selective JAK-inhibitor Tofacitinib in early onset of IVD degeneration and to analyze their capability to maintain disc homeostasis within our recently established degenerative and pro-inflammatory intervertebral disc organ culture model.

## Materials and Methods

### IVD Dissection and Organ Culture

**Figure 1:**
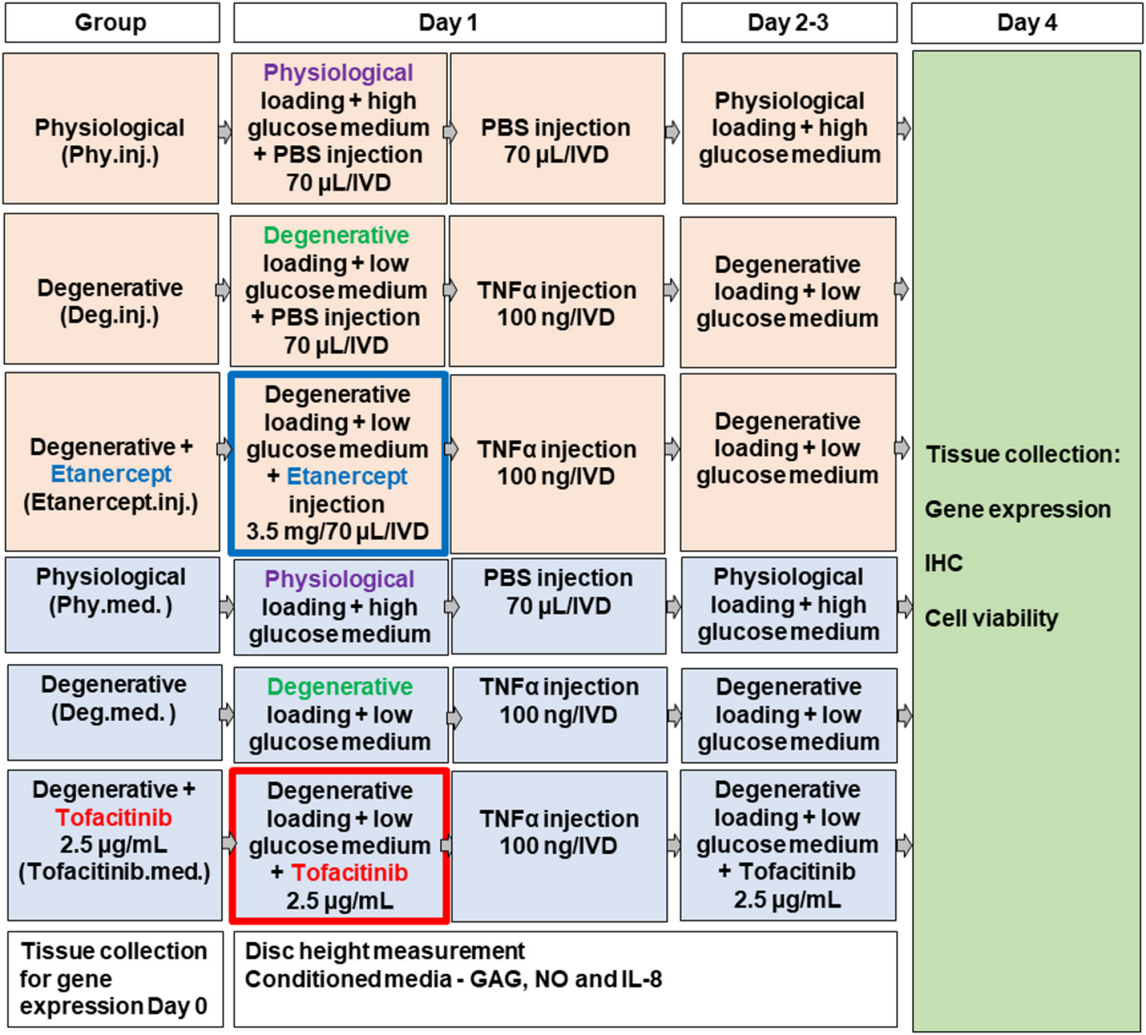
Scheme of experimental setup.

IVD dissection and organ culture were performed as described before (Lang et al., 2018). Experiments were performed using 16 bovine tails (6–12 months old) obtained from local abattoirs. IVDs were harvested and distributed among 3 experimental groups per tail (Figure 1). One disc from each tail was harvested as Day0 control. Discs from the same tail were randomly distributed among the different groups for equivalent distribution of dimensions. Initial average disc height was 11.02 ± 1.21 mm for Tofacitinib and 10.55 ± 1.35 mm for Etanercept experiment sets. The average diameter was 16.73 ± 2.42 and 16.76 ± 1.95 mm for Tofacitinib and Etanercept experiments, respectively. After removal of soft tissue, a band saw (Exakt Apparatebau, Norderstedt, Germany) was used to obtain single units with intact endplates. Capillary blood residues in the bony endplates were removed with a Pulsavac jet-lavage system (Zimmer, Warsaw, IN, USA). IVDs were initially washed with 10% Penicillin/Streptomycin (Pen/Strep) in phosphate-buffered saline (PBS, Sigma–Aldrich, St. Louis, MO, USA) for 15 min, then in 1% Pen/Strep (Sigma–Aldrich) for another 1 min. Hereafter, IVDs were incubated at 37°C, 85% humidity and 5% CO_2_ under free swelling conditions in six-well plates containing Dulbecco's Modified Eagle Medium (DMEM, Sigma–Aldrich) supplied with 2% fetal calf serum (FCS), 1% Pen/Strep, 1% ITS+ Premix (Discovery Labware, Inc., Bedford, MA, USA), 50 μg/mL ascorbate-2-phosphate (Sigma–Aldrich) and 50 μg/mL Primocin (InvivoGen, San Diego, CA, USA).

### IVD Organ Culture Under Different Dynamic Loading and Nutrient Conditions

The procedure for disc harvesting and preparation, as well as the bioreactor used in this study, were the same as described previously (Lang et al., 2018). For this study, a bioreactor system was used to mimic relevant loading conditions of cultured IVDs. IVDs were placed in custom-made chambers filled with 4.5 mL IVD culture medium and were loaded for 4 days within a custom made bioreactor system under 2 different culture conditions (Figure 1) (Lang et al., 2018): (1) physiological cyclic sinusoidal loading (0.02–0.2 MPa; 0.2 Hz; 2 h/day) and high glucose DMEM (4.5 g/L) for **Phy** group, and (2) detrimental cyclic sinusoidal loading (0.32–0.5 MPa; 5 Hz; 2 h/day) and low glucose DMEM medium (2 g/L) for **Deg** group. The polycarbonate bioreactor was designed for the application of uniaxial loading and contained two porous plates. The discs were kept centered in the bioreactor by always maintaining a compressive load in order to ensure contact of bony endplates to the porous plates (Gantenbein et al., 2006; Junger et al., 2009). The entire system was maintained in an incubator kept at 37°C and 5% CO_2_ and ambient O_2._ Four bioreactor chambers were loaded with 1 disc each and the remaining discs were used as fresh d0 controls. The bioreactor system consists of 4 units, with each bioreactor unit independently pneumatically actuated (dSpace data acquisition and control card, dSPACE GmbH, Paderborn, Germany; Matlab-Simulink, MathWorks, Inc., Natick, MA, USA) under force control (load cell, Burster, Gernsbach, Germany, type 8432) (Gantenbein et al., 2006; Junger et al., 2009).

Between loading cycles, IVDs were cultured for free swelling recovery in six-well plates containing corresponding high or low glucose DMEM. Our previous study has shown that combination of 4 days of detrimental culture and single injection of TNF-α significantly upregulated the expression of key proinflammatory and catabolic genes, by mimicking the IVD microenvironment during the early phase of disc degeneration (Lang et al., 2018). Therefore, 100 ng recombinant human TNF-α (R&D systems, Zug, Switzerland) within 70 μl PBS/IVD was injected into the nucleus pulposus (NP) tissue of the Deg and Drug groups after the first dynamic loading cycle on day 1 to trigger inflammation. Control groups received 70 μL PBS which was shown not to influence cell phenotype and viability (Lang et al., 2018).

### Anti-inflammatory Therapy via Etanercept or Tofacitinib

To investigate the anti-inflammatory and anti-catabolic effects of Etanercept and Tofacitinib, either TNF-α-inhibitor Etanercept (**Etanercept.inj**.) or the selective JAK3-inhibitor Tofacitinib (**Tofacitinib.med**.) were administered to the Drug group (Figure 1). Etanercept (3.5 mg/70 μl, Enbrel®, Pfizer, New York, NY, USA) was injected into the center of the NP with an 30G insulin needle after the first loading cycle followed by TNF-α injection 5 min later. Within the same experiment sets, 70 μl PBS was injected to Phy.inj. and Deg.inj. groups serving as positive or negative controls. The selected concentration of Etanercept at 3.5 mg per bovine caudal IVD (~0.35 mg/mL) was based on literature. It has been reported that in a rat animal model, dosages of 0.1 or 1 mg of Etanercept were required to show appropriate effects on pain-related peptide expression in lumbar discs after intradiscal injection (Inage et al., 2016). A clinical study showed that single intradiscal administration of 10 mg Etanercept on patients with LBP could alleviate pain levels for 8 weeks after follow-up (Sainoh et al., 2016). Furthermore, Caliskan and coworkers investigated the effect of Etanercept on primary cell cultures (first-passage cultures of NP and AF cells) from intact human IVD tissue at doses of 0.1, 0.25, 0.5, 1, and 2 mg/mL (Caliskan et al., 2019). The Etanercept doses of >0.5 mg/mL were found to have cytotoxic effects. Hence the Etanercept concentration in the current study at 0.35 mg/mL is speculated to have no cytotoxic effect on disc cells. Olmarker et al. studied whether Etanercept prevents nucleus pulposus-induced functional and structural nerve root injury in a porcine experimental model by assessment of nerve conduction velocity and histology (Olmarker and Rydevik, 2001). Etanercept was applied at a dose of 12.5 mg by subcutaneous injection into pigs of ~25 kg body weight during surgery followed by a second application 3 days later. Etanercept was found to reduce nerve fiber injury, intracapillary thrombus formation, intraneural edema formation, and to prevent a decrease of nerve conduction velocity. The average disc height for human lumbar IVDs was reported to be 10 mm and average disc diameter to be 35 mm (Bergknut et al., 2012). The average initial disc height of the bovine IVDs in this experiment was 10.55 mm and the average disc diameter was 16.76 mm. Thus, the disc volume (V = π · r^2^ · h) of bovine caudal IVDs used in this study is around 2.34 cm3, and the disc volume of human lumbar IVDs is around 9.62 cm3, revealing a volume ratio of ~1:4. Since 10 mg Etanercept injection into human lumbar IVDs was effective to relieve pain in patients according to Sainoh et al. (2016). The dose of 3.5 mg Etanercept chosen in this study is thus within an assumed therapeutic range in our model, considering the smaller distribution volume of the bovine IVDs.

For Tofacitinib, a dose of 10 mg Tofacitinib p.o. twice a day was proposed for the treatment of rheumatoid arthritis, with a body fluid volume of around 87 L and oral bioavailability of 74% (Fleischmann et al., 2016). Additionally, Suzuki and coworkers recently evaluated the anti-inflammatory effect of 100 mmol/L Tofacitinib (31240 μg/mL) on primary rat AF cells supplemented with the IL-6 *in vitro* and observed significant suppression of catabolic effects of IL-6 (Suzuki et al., 2017; NLM National Center for Biotechnology Information, 2020). In the current study, a concentration of 250 ng/mL tofacitinib citrate (Selleckchem, Munich, Germany) was first chosen to be added to the culture medium following every medium change to simulate a systemic application comparable to clinical application of the drug. Hence, the amount of a usual oral dose of 100 mg distributes to a serum concentration of 250 ng/mL. This concentration was based on the highest dose used in clinical study (Krishnaswami et al., 2011). A 10 times higher concentration at 2.5 μg/mL was also tested to investigate the dose-response. Results of the pilot study showed that the higher dose of 2.5 μg/mL has a stronger trend to reduce the gene expression of proinflammatory markers in the NP tissue (Supplementary Figure 1). Thus, a concentration of 2.5 μg/mL tofacitinib citrate was selected in the main study. Tofacitinib citrate stock solution was dissolved in dimethyl sulfoxide (DMSO) at a concentration of 5 mg/mL. In the Deg group 0.05% DMSO was added to the culture medium as vehicle control. Addition of 0.05% DMSO did not cause any dysregulation on the inflammatory, catabolic, and anabolic markers in the vehicle control group.

### Disc Height Change

Disc height was measured twice per day, after free swelling culture overnight and after each dynamic loading, to assess the extent of biomechanical alterations within cultured IVDs. The disc height loss and recovery percentage were assessed by calculation of the average of two measurements. Values were normalized to the initial dimensions right after dissection.

### Gene Expression in IVD Tissue

RT-qPCR was performed to investigate gene expression levels of key proinflammatory, catabolic, and pain related markers as demonstrated before (Lang et al., 2018). From each IVD sample, 150 mg of NP and AF tissue were digested with 2 mg/mL pronase for 1 h at 37°C, then flash frozen, pulverized, and homogenized using liquid nitrogen and a TissueLyser (Qiagen, Venlo, Netherlands) (Caprez et al., 2018). Total mRNA was extracted using TRI Reagent (Molecular Research Center, Cincinnati, OH, USA) and reverse transcription was performed with a SuperScript VILO cDNA Synthesis Kit (Life Technologies, Carlsbad, CA, USA). Quantitative real-time PCR was performed using the Quant Studio Flex 6 instrument (Life Technologies) (Kazezian et al., 2017; Li et al., 2017). Custom designed bovine primers and TaqMan™ probes from Microsynth (Balgach, Switzerland; Supplementary Table 1) were used for amplification of Interleukin-1b (IL-1β), Interleukin-6 (IL-6), Matrix Metalloprotease (MMP) 1, 3, and 13. For amplification of ribosomal protein large P0 (RPLP0, Bt03218086_m1), IL-8 (Bt03211906_m1), NGF (Bt03817604_s1), and COX2 (Bt03214492_m1), gene expression assays from Applied Biosystems (Life Technologies) were used. Relative quantification of the target mRNA was performed using comparative Ct method with RPLP0 as endogenous control (Lopa et al., 2016).

### Medium Analysis

Conditioned medium was collected for analysis of released matrix components and mediators daily before and after loading for further analysis. Glycosaminoglycan (GAG) content and levels of nitric oxide (NO) in the conditioned medium were determined using a modified DMMB method and a Griess Reagent Kit (Promega, Madison, WI, USA) (Farndale et al., 1986; Lang et al., 2018). The Griess Reagent Kit is based on the chemical reaction which utilizes sulfanilamide and N-1-napthylethylenediamine dihydrochloride (NED) under acidic (phosphoric acid) conditions. This system detects ${\text{NO}}_{2}^{-}$ in various liquids. For the ${\text{NO}}_{2}^{-}$ measurement, a nitrite standard reference curve was prepared for each assay. Hereafter, 50 μl of each sample was added to wells in duplicates. Fifty microliter of the Sulfanilamide Solution was dispensed to all experimental samples and wells containing the dilution series for the Nitrite Standard reference curve following incubation for 10 min at room temperature, protected from light. Additionally, 50 μl of the NED Solution was dispensed to all wells following incubation for 10 min, protected from light. Finally, the absorbance was measured in a plate reader (Victor3 Micro Plate Reader, PerkinElmer, Waltham, MA, USA) with a filter between 520 and 550 nm. For the modified DMMB method, a GAG standard reference curve was prepared for each assay utilizing 1 mg/ml chondroitinsulfate (Chondroitin 4-sulfate sodium salt from bovine trachea, mixture of isomers, Sigma-Aldrich) in distilled H_2_O. Hereafter, 50 μl of each sample was added to wells in duplicates following the supplementation of 200 μl of DMMB color reagent [16 mg DMMB (1,9-Dimethyl-methylene blue, Sigma-Aldrich) in 1 L water containing 3.04 g glycine and 2.37 g NaCl)] to each well. Absorbance was read in a plate reader (Victor3 Micro Plate Reader) at 535 nm. Interleukin 8 (IL-8) protein content was measured with bovine IL-8 ELISA kits (Kingfisher Biotech, St. Paul, MN, USA).

### Histology

After 4 days of culture, endplates were removed from one side and whole IVDs were snap frozen in Tissue Freezing Medium® (Leica Biosystems, Nussloch, Germany). Transverse sections (10 μm) were obtained using a cryostat (Microm, Dreieich, Germany). Immunohistochemistry (IHC) was performed to investigate protein expression of IL-1β, IL-6, IL-8, and collagen type II within the disc tissue. Cryosections were fixed in 70% and 100% methanol for 10 min each, then left airdry overnight. The slides were rinsed in deionized water and washed in 99% methanol with 0.3% H_2_O_2_ solution for 30 min. After blocking with goat serum (Vector Laboratories, Burlingame, CA, USA), the sections were probed with IL-1β (Kingfisher Biotech, KP1109B-100, 2 μg/mL), IL-6 (Kingfisher Biotech, KP0652B-100, 1 μg/mL) or IL-8 (Kingfisher Biotech, PB0273B-100, 5 μg/mL) antibodies at 4°C overnight. The sections were then incubated with a biotinylated goat-anti-rabbit secondary antibody (Vector Laboratories, #BA-1000) for IL-1β and IL-8, or goat-anti-chicken secondary antibody (Vector Laboratories, #BA-9010) for IL-6, followed by incubation with avidin-biotin-peroxidase (Vectastain Elite ABC kit, Vector Laboratories), and ImmPACT®DAB Peroxidase (HRP) Substrate (Vector Laboratories). For collagen type II IHC staining, after enzyme treatment with 1 U/mL hyaluronidase (Sigma-Aldrich), non-specific binding sites were blocked with horse serum (Vector Laboratories) for 1 h at RT. The primary antibody (CIIC1, 5 μg/mL, Developmental Studies Hybridoma Bank, University of Iowa, Iowa City, IA, USA) was incubated for 30 min at RT and detected using a secondary biotinylated anti-mouse antibody (Vector Laboratories) followed by incubation with avidin-biotin-peroxidase complex, and ImmPACT®DAB Peroxidase (HRP) Substrate. Negative control sections were incubated without the primary antibody. As described previously, cell viability was assessed using lactate dehydrogenase (LDH) staining in 40% polypep solution and ethidium homodimer-1 staining (EthD-1, 1 μg/mL). The blue or blue/red staining indicates living cells, and red only staining indicates dead cells (Li et al., 2016b; Lang et al., 2018). For both IHC and LDH/ EthD-1 staining, 2 IVDs per group were analyzed. For each IVD sample, 4 images at each predefined anatomic region (NP, inner AF, and outer AF) were analyzed. In IL-1β, IL-6 and IL-8 IHC images, the percentage of positively stained cells was analyzed using Axioplan software (Zeiss, Oberkochen, Germany). In collagen type II IHC images, the staining optical density (OD)/area value was analyzed using Image-Pro Plus 6.0 software (Media Cybernetics, Rockville, MD, USA). In LDH/EthD-1 staining images, the percentage of alive cells was analyzed using Axioplan software (Zeiss).

### Statistical Analysis

GraphPad Prism 7 software (GraphPad Software, Inc., La Jolla, CA, USA) was used for statistical analysis. D'Agostino-Pearson omnibus normality test was performed to assess if data were normally distributed. For data that were normally distributed, unpaired *t*-test was used to determine differences between two groups; one-way ANOVA was used to determine differences between three or more groups. For data that were not normally distributed, Mann–Whitney *U*-test was used to determine differences between two groups; Kruskal Wallis test was used to determine differences between three or more groups. A *p* < 0.05 was considered statistically significant.

## Results

### Gene Expression

**Figure 2:**
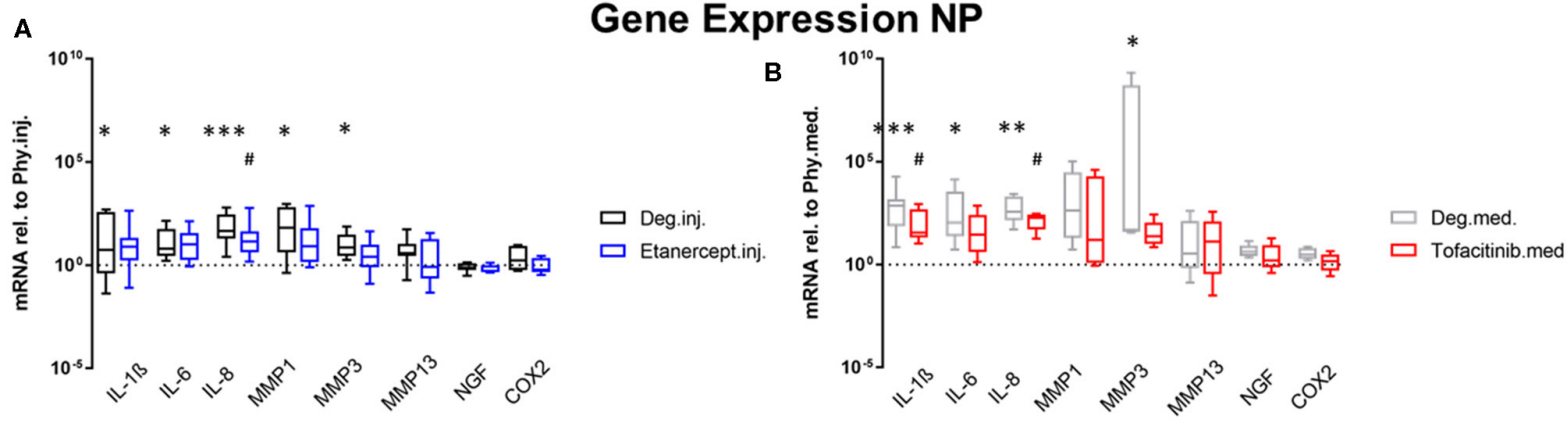
Gene expression levels of NP tissue. Gene expression levels of NP tissue after 4 days of culture under Deg culture condition, with or without **(A)** Etanercept intradiscal injection. **(B)** Tofacitinib application into the medium, normalized to Phy group. *n* = 8, *p < 0.05, **p < 0.01, ***p < 0.001 Deg vs. Phy, ^#^*p* < 0.05 Drug vs. Phy, min to max (bottom and top bar) with interquartile range (middle box). NP, Nucleus pulposus; IL, Interleukin; MMP, Matrix metalloproteinases; NGF, Nerve growth factor; COX-2, Cyclooxygenase-2.

**Figure 3:**
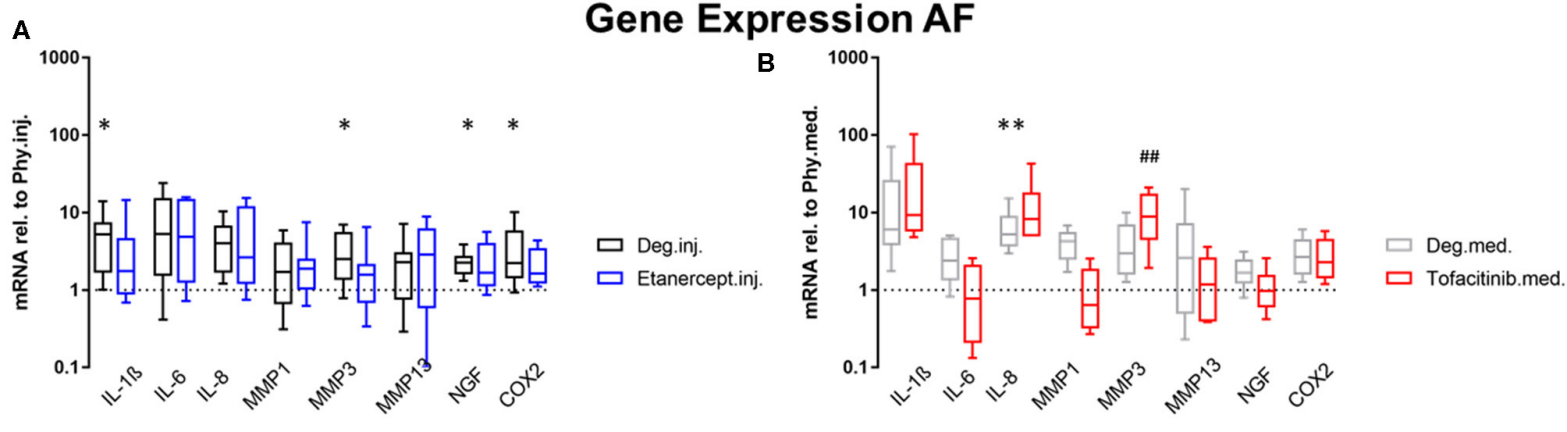
Gene expression levels of AF tissue. Gene expression levels of AF tissue after 4 days of culture under Deg culture condition, with or without **(A)** Etanercept intradiscal injection. **(B)** Tofacitinib application into the medium, normalized to Phy group. *n* = 8, *p < 0.05, **p < 0.01 Deg vs. Phy, ^##^*p* < 0.01 Drug vs. Phy, min to max (bottom and top bar) with interquartile range (middle box). AF, Annulus fibrosus; IL, Interleukin; MMP, Matrix metalloproteinases; NGF, Nerve growth factor; COX-2, Cyclooxygenase-2.

Gene expression levels of NP and AF tissues were normalized to Phy group. According to the results of the D'Agostino-Pearson omnibus normality test, the gene expression data were not non-normally distributed. Therefore, Kruskal Wallis test was used to detect the differences between the 3 groups: Phy, Deg, and Drug. Deg.inj. culture condition upregulated the expression of catabolic enzymes MMP1 (*p* < 0.05) and MMP3 (*p* < 0.05) in NP tissue (Figure 2). Deg.med. culture condition upregulated the expression of catabolic enzymes MMP3 (*p* < 0.05) in NP tissue (Figure 2). Detrimental culture conditions also resulted in a strong inflammatory response, indicated by a significant upregulation of proinflammatory markers, including IL-1β in NP (Deg.inj. and Deg.med.) and AF tissue (Deg.inj., *p* < 0.05), IL-6 in NP tissue (Deg.inj. and Deg.med., *p* < 0.05) and IL-8 in NP (Deg.inj. and Deg.med, *p* < 0.05) and AF tissue (Deg.med, *p* < 0.05) (Figures 2, 3). COX2 (*p* < 0.05) and NGF (*p* < 0.05) showed a significant upregulation under Deg.inj. culture conditions in the AF (Figure 3). Overall, detrimental culture conditions, implying degenerative loading combined with the pro-inflammatory stimulus TNF-α, featured stronger effects in the NP than in AF tissue.

Compared to the Deg.inj. group, Etanercept treatment partially prevented the upregulation of IL-1β, IL-6, IL-8, MMP1, and MMP3 in the NP (Figure 2) and IL-1β, MMP3, COX2, and NGF in the AF (Figure 3), resulting in expression levels comparable with the Phy.inj. group. Compared to the Deg.med. group, Tofacitinib therapy partially prevented the increase of IL-1β, IL-6, IL-8, and MMP3 in the NP (Figure 2).

### GAG, NO, and IL-8 Protein Release in Medium

**Figure 4:**
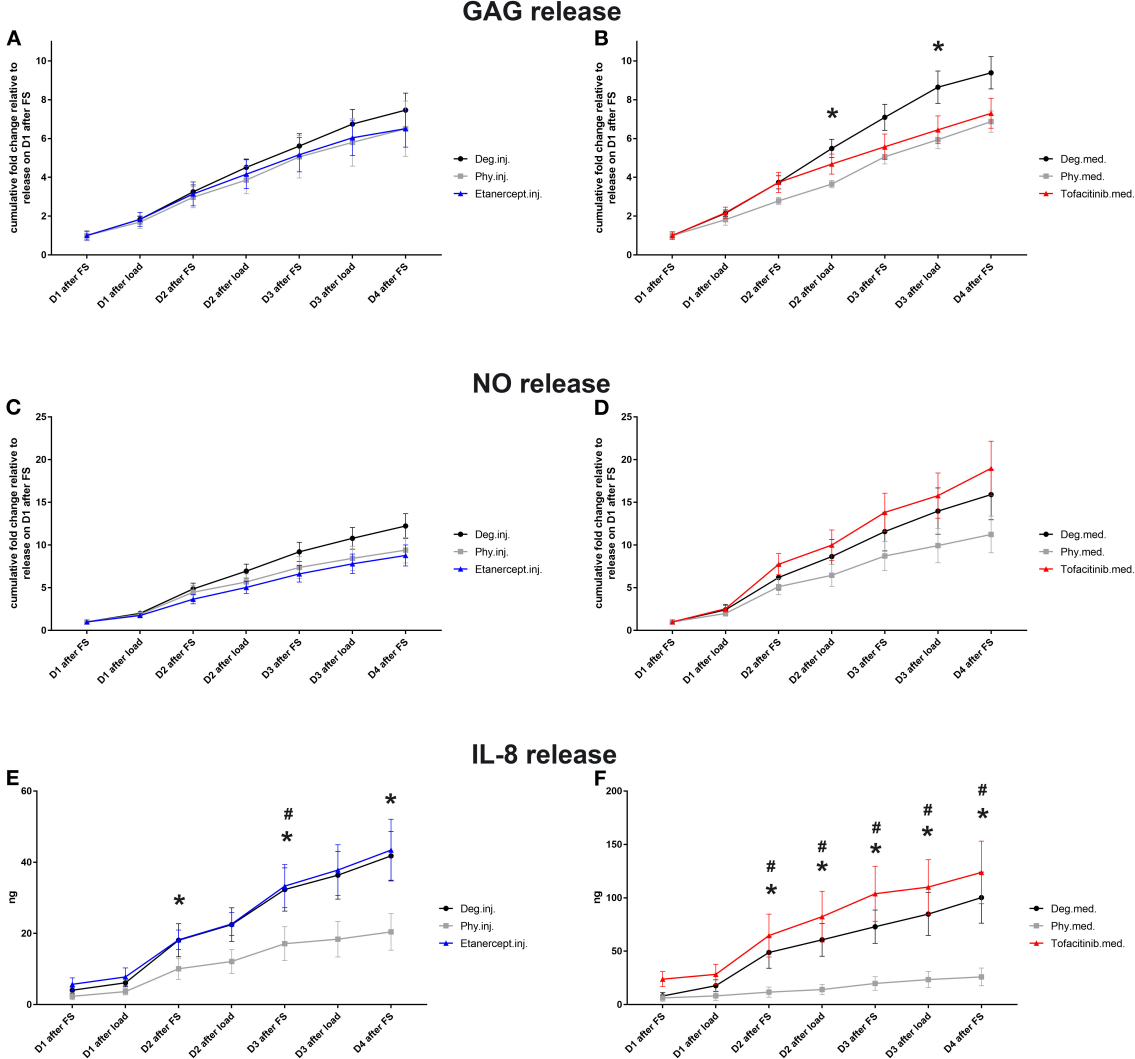
GAG **(A,B)**, NO **(C,D)**, and IL-8 release **(E,F)** in IVD culture medium. Cumulative release of GAG, NO, and IL-8 into IVD conditioned medium during 20 h of free swelling culture (FS) and 2 h of dynamic loading (load); D, day; Means ± SEM, *n* = 8; *p < 0.05 Phy vs. Deg; ^#^*p* < 0.05 Phy vs. Drug; GAG, glycosaminoglycan; NO, nitric oxide.

Conditioned IVD medium was collected for investigation of GAG, NO, and IL-8 protein release (*n* = 8). The cumulative GAG release was normalized to the volume of each disc on day 0 after dissection, which was then normalized to the value of respective group on day 1 during 20 h of free swelling culture (Figures 4A,B) GAG release under Deg.med. culture condition was significantly enhanced compared to Phy.med. culture condition on Day 2 and Day 3 after loading (*p* < 0.05). Deg.inj. condition also caused an enhanced release of GAG compared to Phy.inj. culture condition. Etanercept and Tofacitinib reduced the GAG loss to the level under physiological culture condition (Figures 4A,B). The cumulative NO release was normalized to the volume of each disc on day 0 after dissection, which was then normalized to the value of the respective group on day 1 during free swelling (Figures 4C,D). An increased NO release was observed under Deg.inj. and Deg.med. culture conditions compared to Phy.inj. and Phy.med. groups. Etanercept reduced the NO release, while Tofacitinib had no effect. Deg.inj. and Deg.med. culture conditions significantly upregulated IL-8 protein release compared to physiological culture conditions (*p* < 0.05) (Figures 4E,F). Furthermore, there was a peak of IL-8 release among all Deg and Drug groups after the TNF-α injection. Cumulation with time revealed an increasing difference of IL-8 release in the Deg groups compared to the Phy groups, indicating a constant increase of proinflammatory cytokines due to Deg treatment. Etanercept and Tofacitinib did not show any reduction effect on IL-8 release.

### IL-1β, IL-6, and IL-8 Protein Expression in IVD Tissue

**Figure 5:**
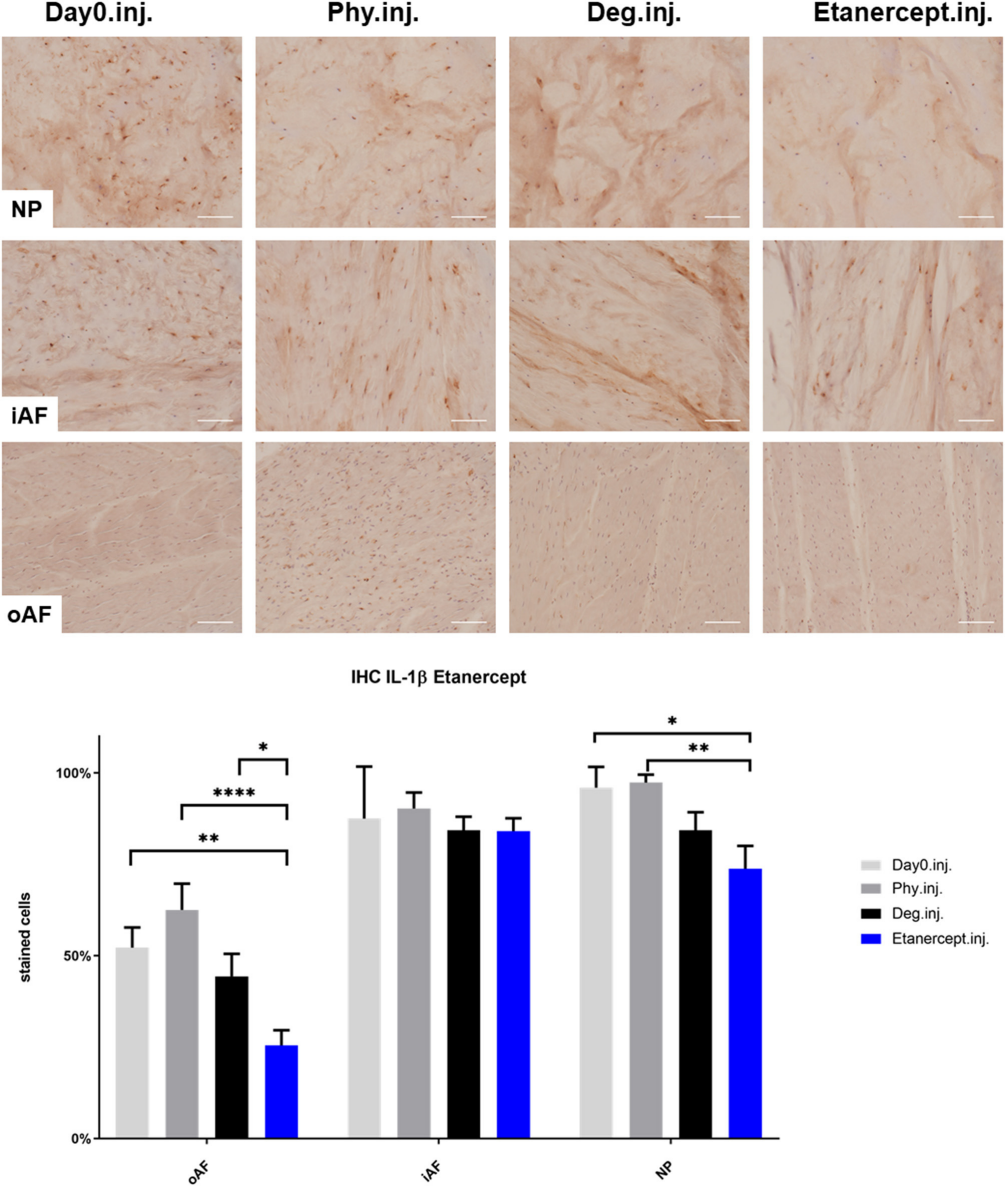
IL-1β immunohistochemistry staining of IVD tissue from the Etanercept experiments. Representative IL-1β IHC image of NP region, inner AF region (iAF) and outer AF (oAF) region from day 0 control samples (Day0.inj.), IVDs cultured under physiological condition on day 4 (Phy.inj.), IVDs cultured under degenerative condition on day 4 (Deg.inj.), and IVDs cultured under degenerative condition and treated with Etanercept on day 4 (Etanercept.inj.). Scale bar: 100 μm. The percentage of positively stained cells were counted, as presented in the bar graph. *n* = 8, Means + SEM, *p < 0.05, **p < 0.01, ****p < 0.0001.

**Figure 6:**
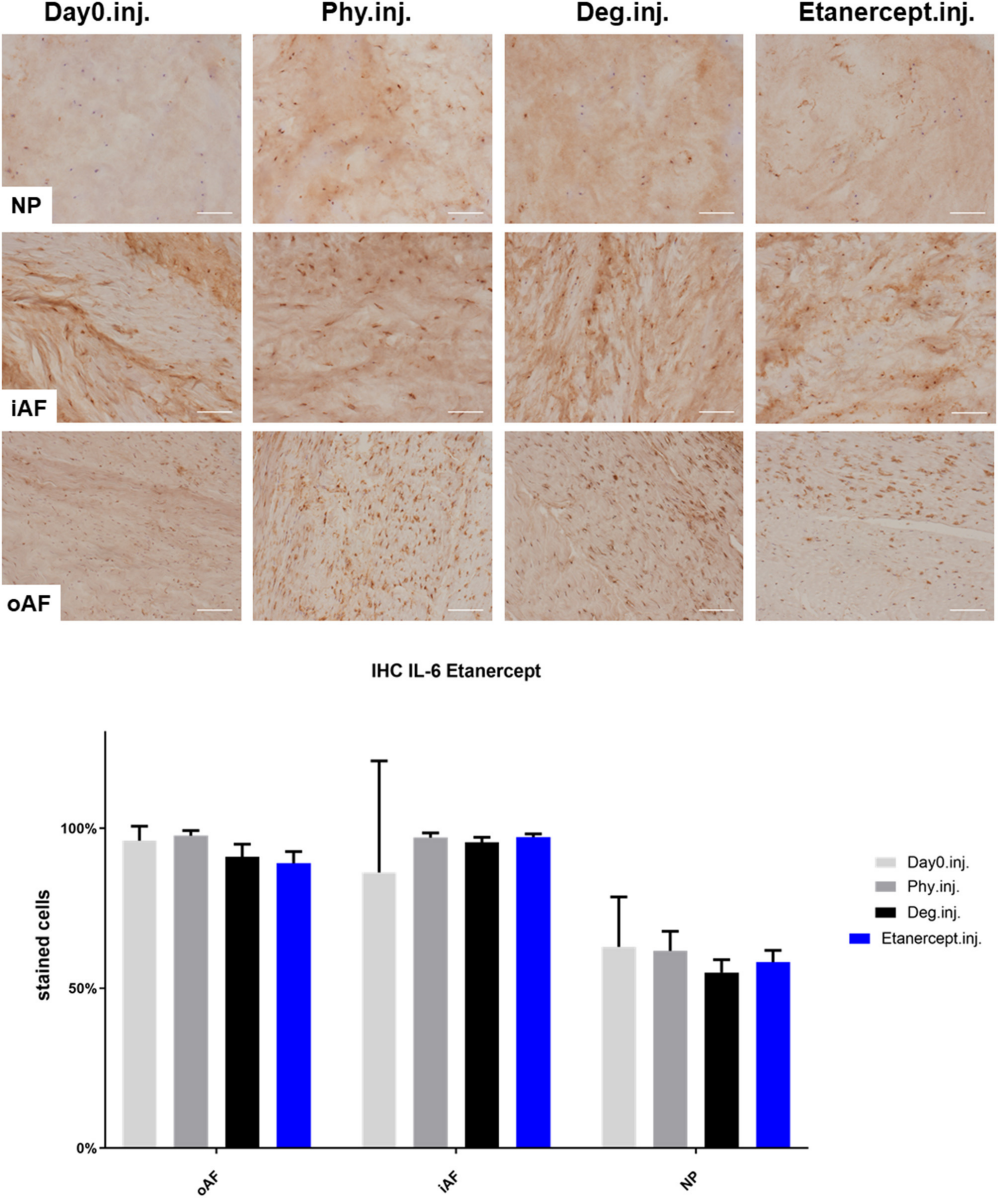
IL-6 immunohistochemistry staining of IVD tissue from the Etanercept experiments. Representative IL-6 IHC image of NP region, inner AF region (iAF) and outer AF (oAF) region from day 0 control samples (Day0.inj.), IVDs cultured under physiological condition on day 4 (Phy.inj.), IVDs cultured under degenerative condition on day 4 (Deg.inj.), and IVDs cultured under degenerative condition and treated with Etanercept on day 4 (Etanercept.inj.). Scale bar: 100 μm. The percentage of positively stained cells were counted, as presented in the bar graph. *n* = 8, Means + SEM.

**Figure 7:**
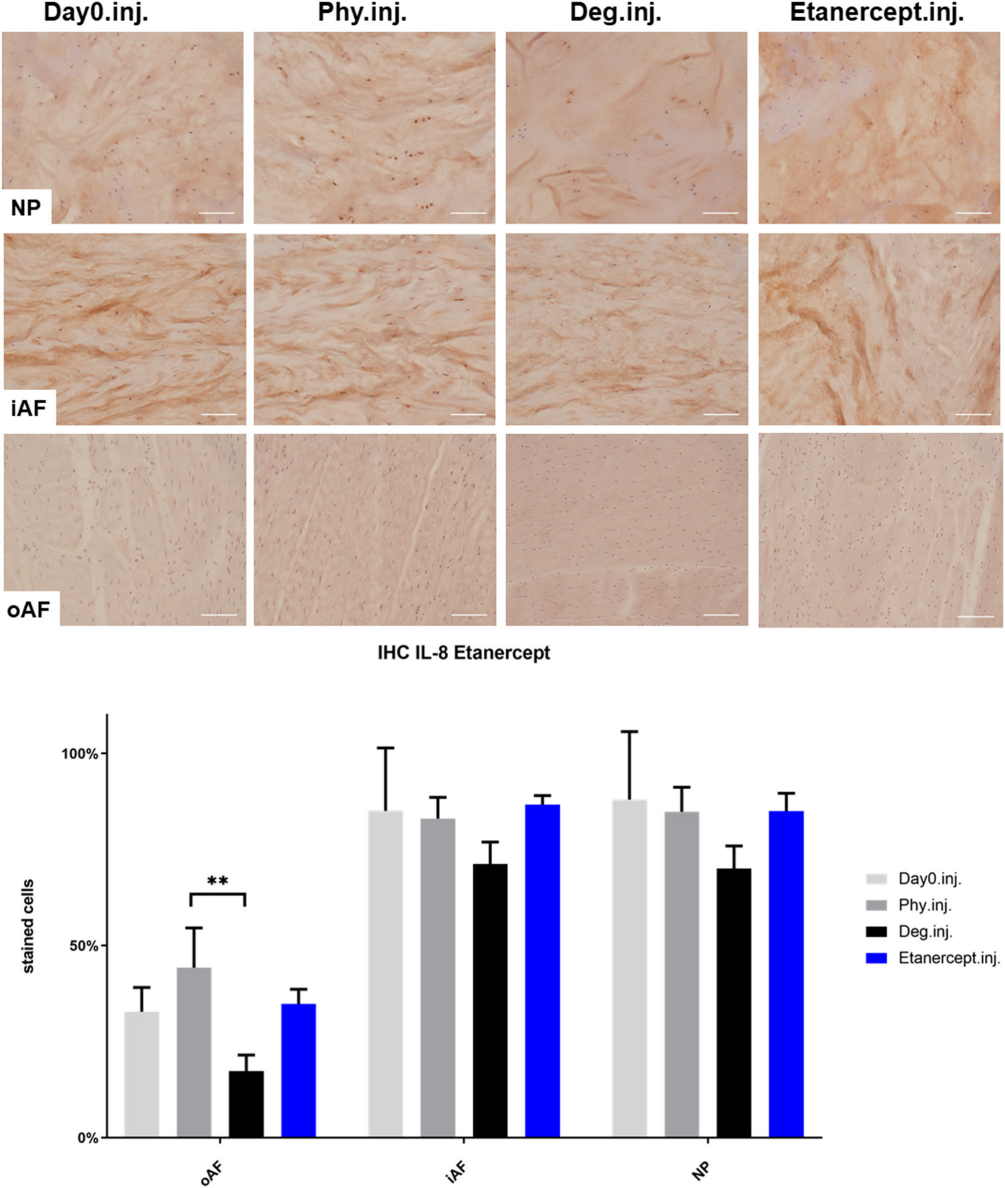
IL-8 immunohistochemistry staining of IVD tissue from the Etanercept experiments. Representative IL-8 IHC image of NP region, inner AF region (iAF) and outer AF (oAF) region from day 0 control samples (Day0.inj.), IVDs cultured under physiological condition on day 4 (Phy.inj.), IVDs cultured under degenerative condition on day 4 (Deg.inj.), and IVDs cultured under degenerative condition and treated with Etanercept on day 4 (Etanercept.inj.). Scale bar: 100 μm. The percentage of positively stained cells were counted, as presented in the bar graph. *n* = 8, Means + SEM, **p < 0.01.

**Figure 8:**
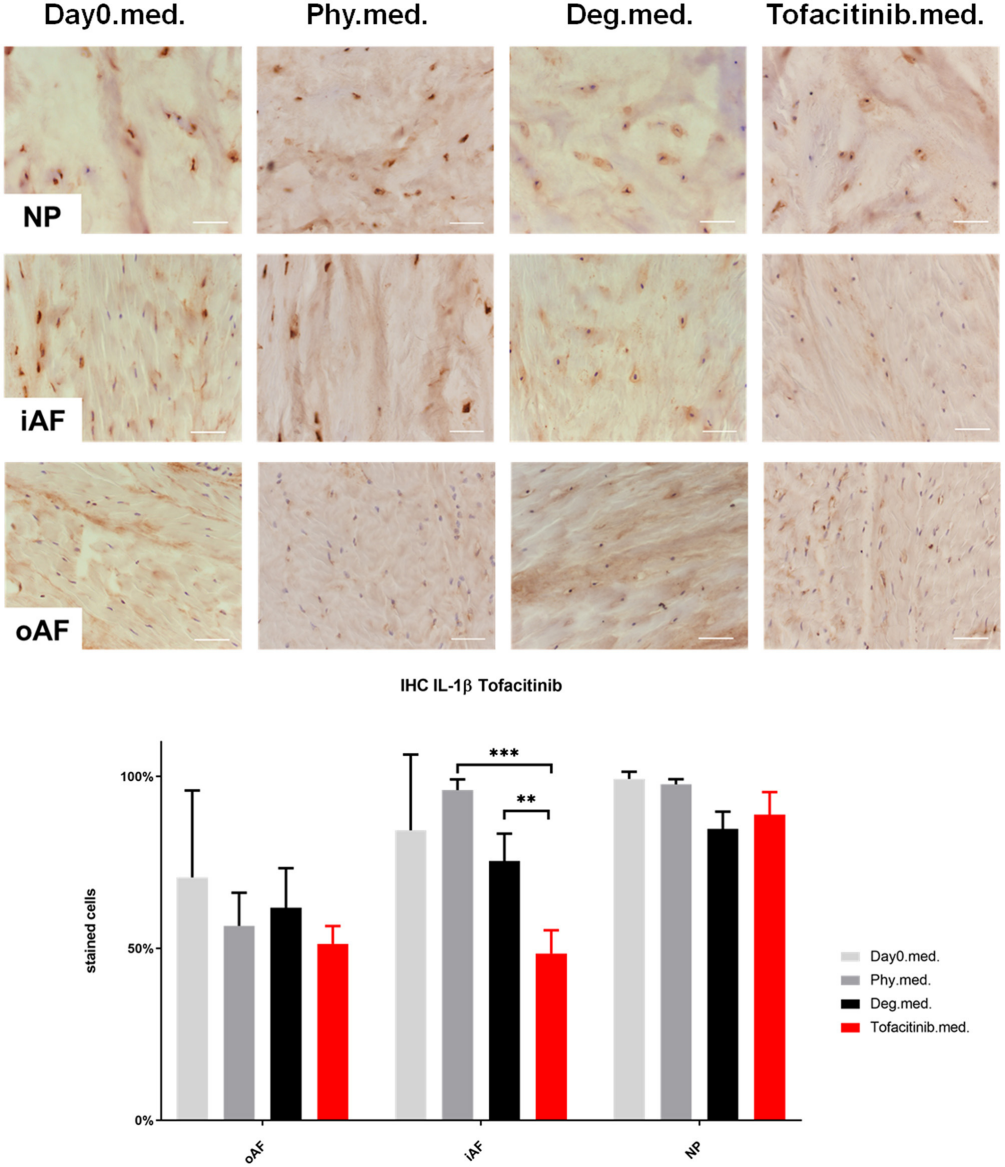
IL-1β immunohistochemistry staining of IVD tissue from the Tofacitinib experiments. Representative IL-1β IHC image of NP region, inner AF region (iAF) and outer AF (oAF) region from day 0 control samples (Day0.med.), IVDs cultured under physiological condition on day 4 (Phy.med.), IVDs cultured under degenerative condition on day 4 (Deg.med.), and IVDs cultured under degenerative condition and treated with Tofacitinib on day 4 (Tofacitinib.med.). Scale bar: 100 μm. The percentage of positively stained cells were counted, as presented in the bar graph. *n* = 8, Means + SEM, **p < 0.01, ***p < 0.001.

**Figure 9:**
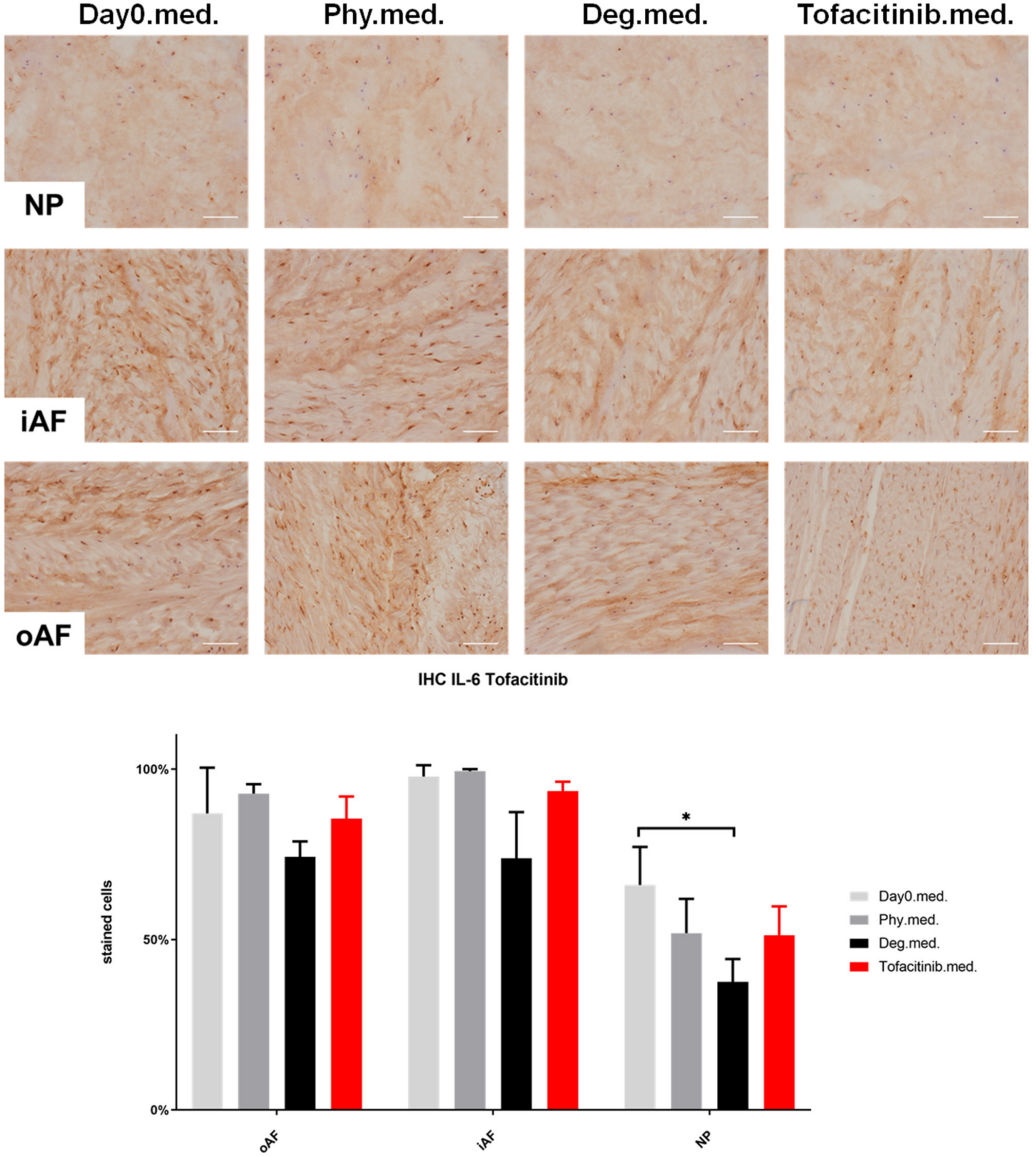
IL-6 immunohistochemistry staining of IVD tissue from the Tofacitinib experiments. Representative IL-6 IHC image of NP region, inner AF region (iAF) and outer AF (oAF) region from day 0 control samples (Day0.med.), IVDs cultured under physiological condition on day 4 (Phy.med.), IVDs cultured under degenerative condition on day 4 (Deg.med.), and IVDs cultured under degenerative condition and treated with Tofacitinib on day 4 (Tofacitinib.med.). Scale bar: 100 μm. The percentage of positively stained cells were counted, as presented in the bar graph. *n* = 8, Means + SEM, *p < 0.05.

**Figure 10:**
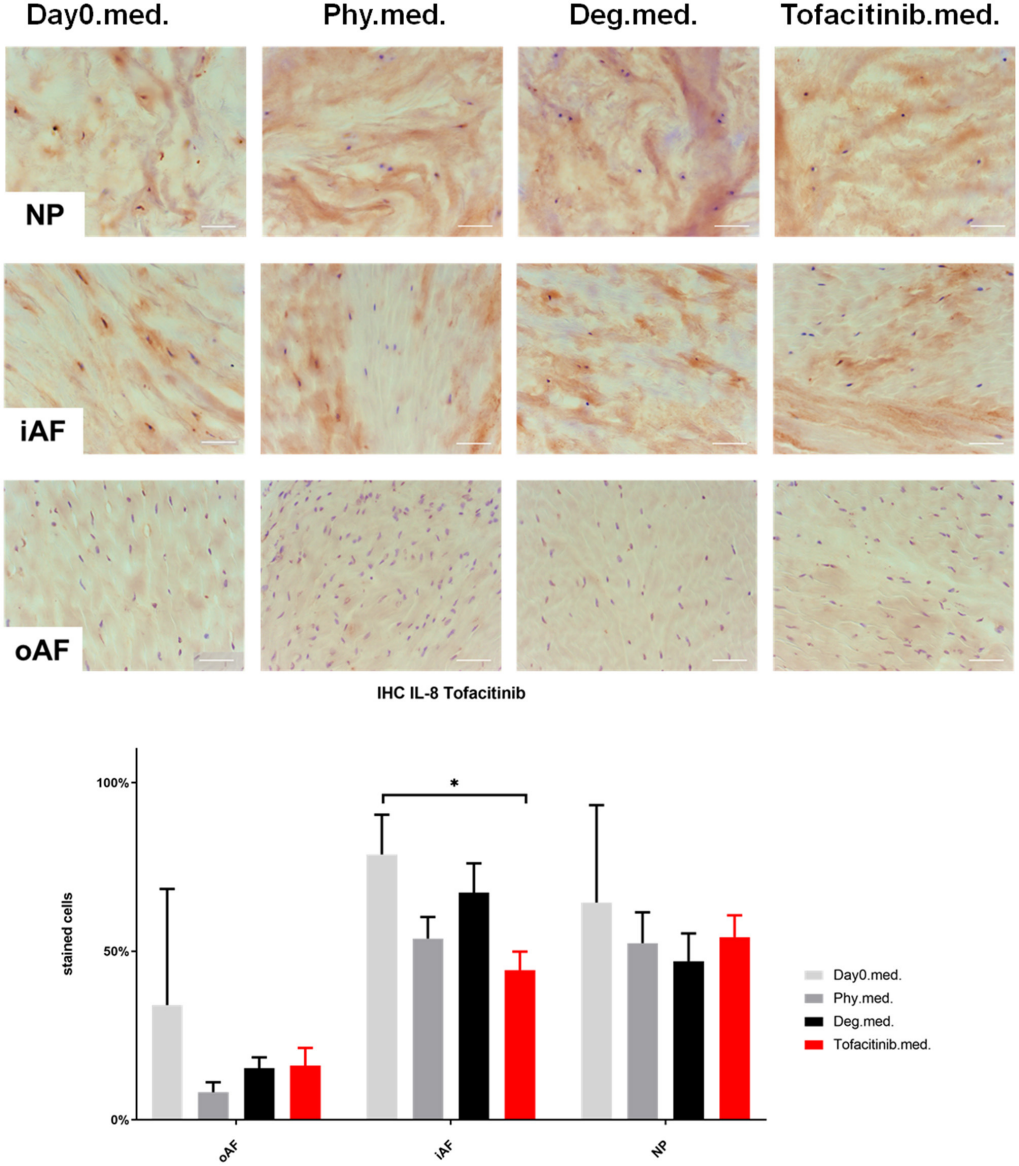
IL-8 immunohistochemistry staining of IVD tissue from the Tofacitinib experiments. Representative IL-8 IHC image of NP region, inner AF region (iAF) and outer AF (oAF) region from day 0 control samples (Day0.med.), IVDs cultured under physiological condition on day 4 (Phy.med.), IVDs cultured under degenerative condition on day 4 (Deg.med.), and IVDs cultured under degenerative condition and treated with Tofacitinib on day 4 (Tofacitinib.med.). Scale bar: 100 μm. The percentage of positively stained cells were counted, as presented in the bar graph. *n* = 8, Means + SEM, *p < 0.05.

IHC was performed to investigate the protein expression of IL-1β, IL-6, and IL-8 within IVD tissue (Figures 5–10). For qualitative analysis, representative IL-1β, IL-6, and IL-8 IHC images were taken from the NP region, inner AF region, and outer AF region from day 0 control samples (Day0.inj.), IVDs cultured under physiological condition on day 4 (Phy.inj./Phy.med.), IVDs cultured under degenerative condition on day 4 (Deg.inj./Deg.med.), and IVDs cultured under degenerative condition and treated either with Etanercept (Etanercept.inj.) or Tofacitinib on day 4 (Tofacitinib.med.). Additionally, the percentage of positively stained cells were counted, as presented in the bar graphs (Figures 5–10). IHC revealed expression of proinflammatory cytokines IL-1β, IL-6, and IL-8 throughout all experimental groups. Trends of differences in the distribution of interleukins among the different IVD tissue types were observable. In general, physiological expression of IL-1β and IL-8 was more abundant in the NP whereas IL-6 featured higher expression levels in the AF. Degenerative culture condition reduced the percentage of IL-8 positively stained cells in the outer AF tissue compared with physiological group (Deg.inj; Figure 7), as well as the IL-6 positively stained cells in the NP tissue compared with day 0 group (Deg.med.; Figure 9). A reduction of IL-1β positively stained cells in the outer AF and NP regions after Etanercept treatment (Figure 5, *p* < 0.05), reduction of IL-1β positively stained cells in the inner AF region following Tofacitinib therapy (Figure 8, *p* < 0.01), as well as reduction of IL-8 positively stained cells in inner AF tissue after Tofacitinib (Figure 10, *p* < 0.05) were observed comparing to at least one of the other groups.

### Collagen Type II Protein Expression in IVD Tissue

**Figure 11:**
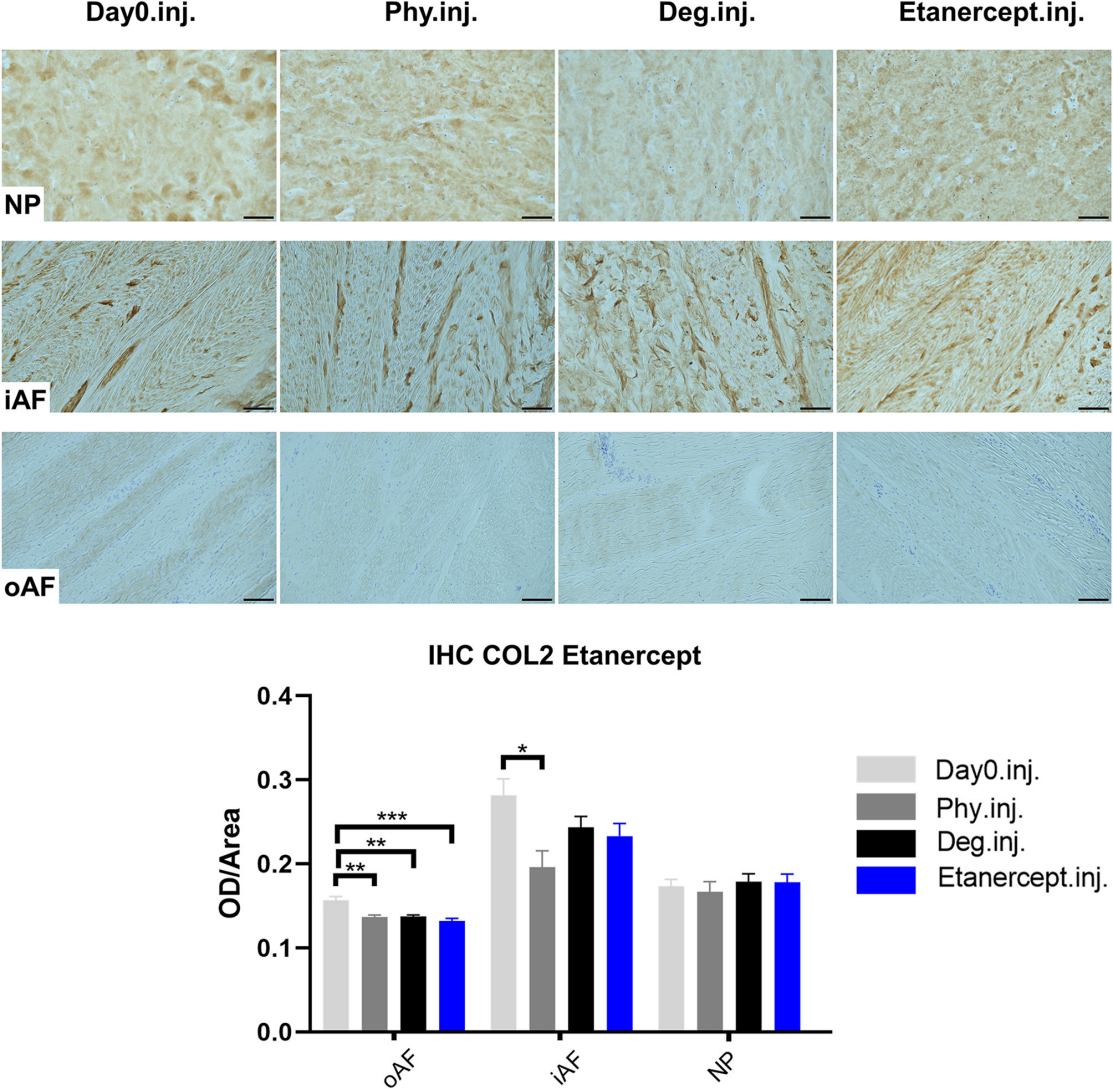
Collagen type II immunohistochemistry staining of IVD tissue from the Etanercept experiments. Representative COL2 IHC image of NP region, inner AF region (iAF) and outer AF (oAF) region from day 0 control samples (Day0.inj.), IVDs cultured under physiological condition on day 4 (Phy.inj.), IVDs cultured under degenerative condition on day 4 (Deg.inj.), and IVDs cultured under degenerative condition and treated with Etanercept on day 4 (Etanercept.inj.). Scale bar: 100 μm. The staining optical density (OD)/area value was analyzed, as presented in the bar graph. *n* = 8, Means + SEM, *p < 0.05, **p < 0.01, ***p < 0.001.

**Figure 12:**
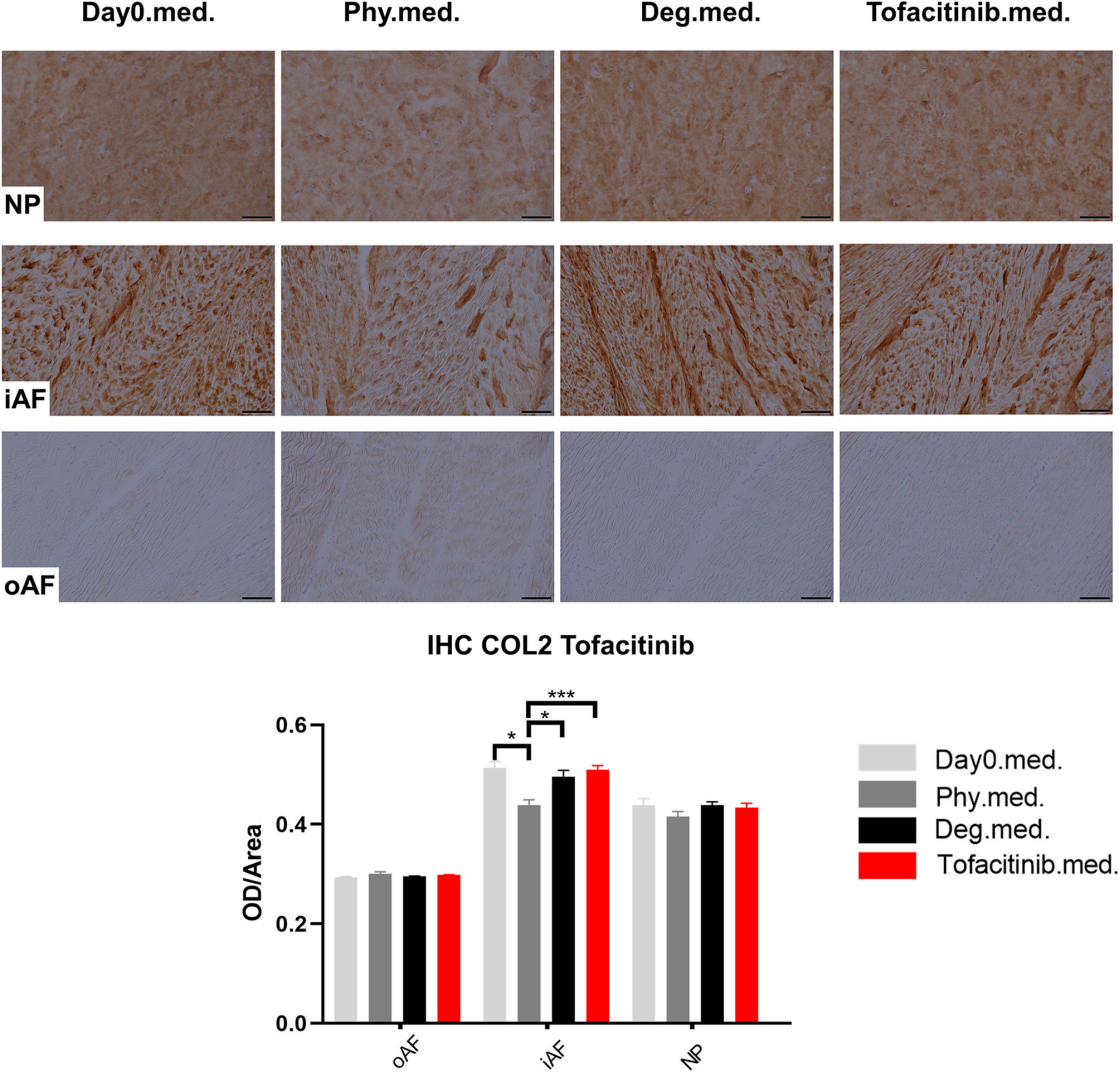
Collagen type II immunohistochemistry staining of IVD tissue from the Tofacitinib experiments. Representative COL2 IHC image of NP region, inner AF region (iAF) and outer AF (oAF) region from day 0 control samples (Day0.med.), IVDs cultured under physiological condition on day 4 (Phy.med.), IVDs cultured under degenerative condition on day 4 (Deg.med.), and IVDs cultured under degenerative condition and treated with Tofacitinib on day 4 (Tofacitinib.med.). Scale bar: 100 μm. The staining optical density (OD)/area value was analyzed, as presented in the bar graph. *n* = 8, Means + SEM, *p < 0.05, ***p < 0.001.

COL2 distribution was revealed by IHC in Figures 11, 12. In NP tissue, COL2 was homogenously distributed in the tissue. The expression in inner AF tissue had a stripe shape, with some AF lamellae stained more intense and some other lamellae with lighter staining. The outer AF tissue showed very weak staining of COL2. After 4 days of culture, COL2 expression in the inner AF tissue of IVDs under Phy condition showed a decrease compared with Day0 healthy controls. The Deg and Drug groups showed a slightly higher COL2 staining intensity compared with the Phy group, while no difference was observed between the Deg and Drug groups.

### Cell Viability in IVD Tissue

**Figure 13:**
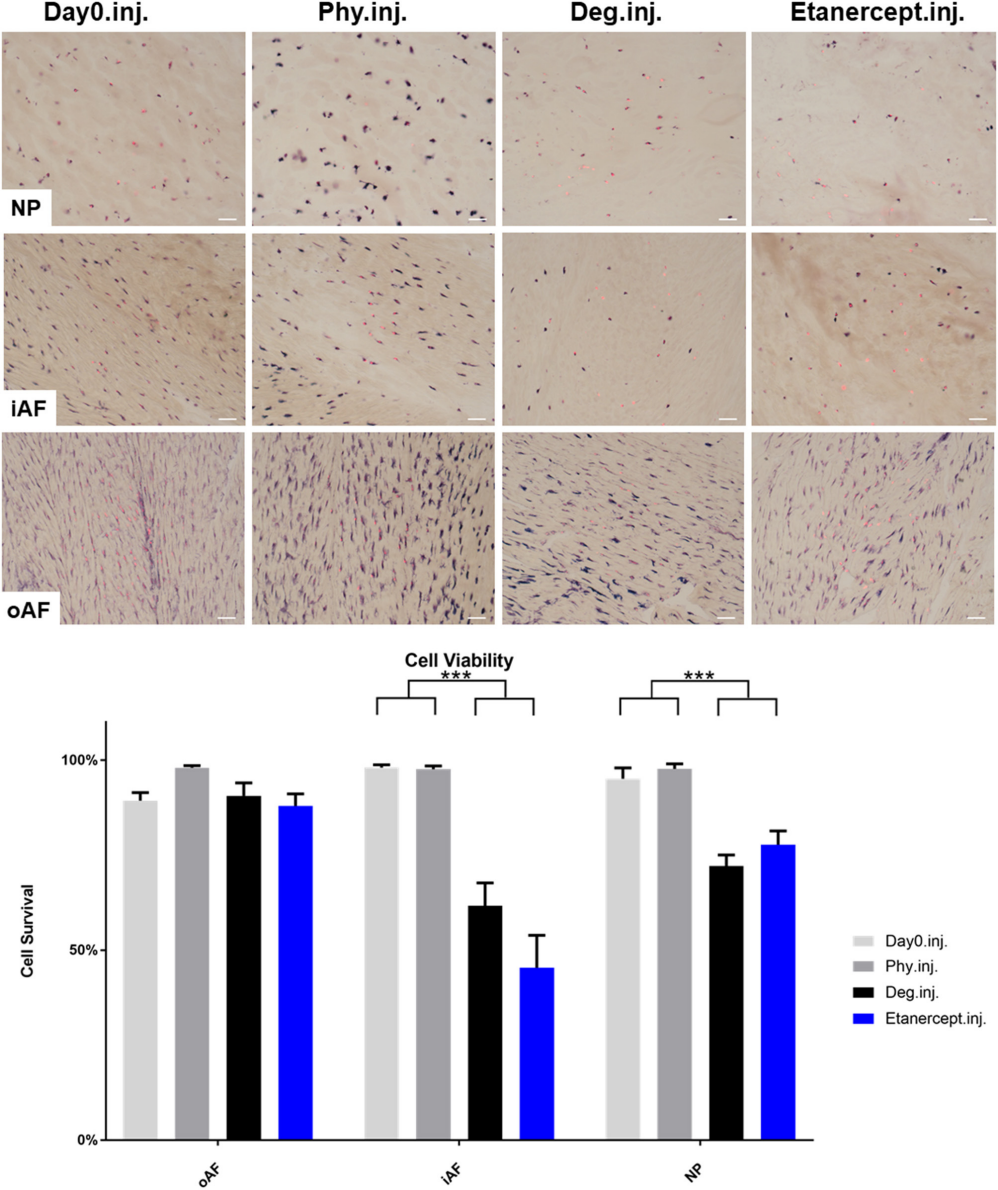
Cell viability of IVD tissue from the Etanercept experiments. Representative LDH/ EthD-1 staining image of NP region, inner AF region (iAF) and outer AF (oAF) region from day 0 control samples (Day0.inj.), IVDs cultured under physiological condition on day 4 (Phy.inj.), IVDs cultured under degenerative condition on day 4 (Deg.inj.), and IVDs cultured under degenerative condition and treated with Etanercept on day 4 (Etanercept.inj.). Scale bar: 50 μm. The percentage of alive cells were counted, as presented in the bar graph. *n* = 8, Means + SEM, ***p < 0.001.

**Figure 14:**
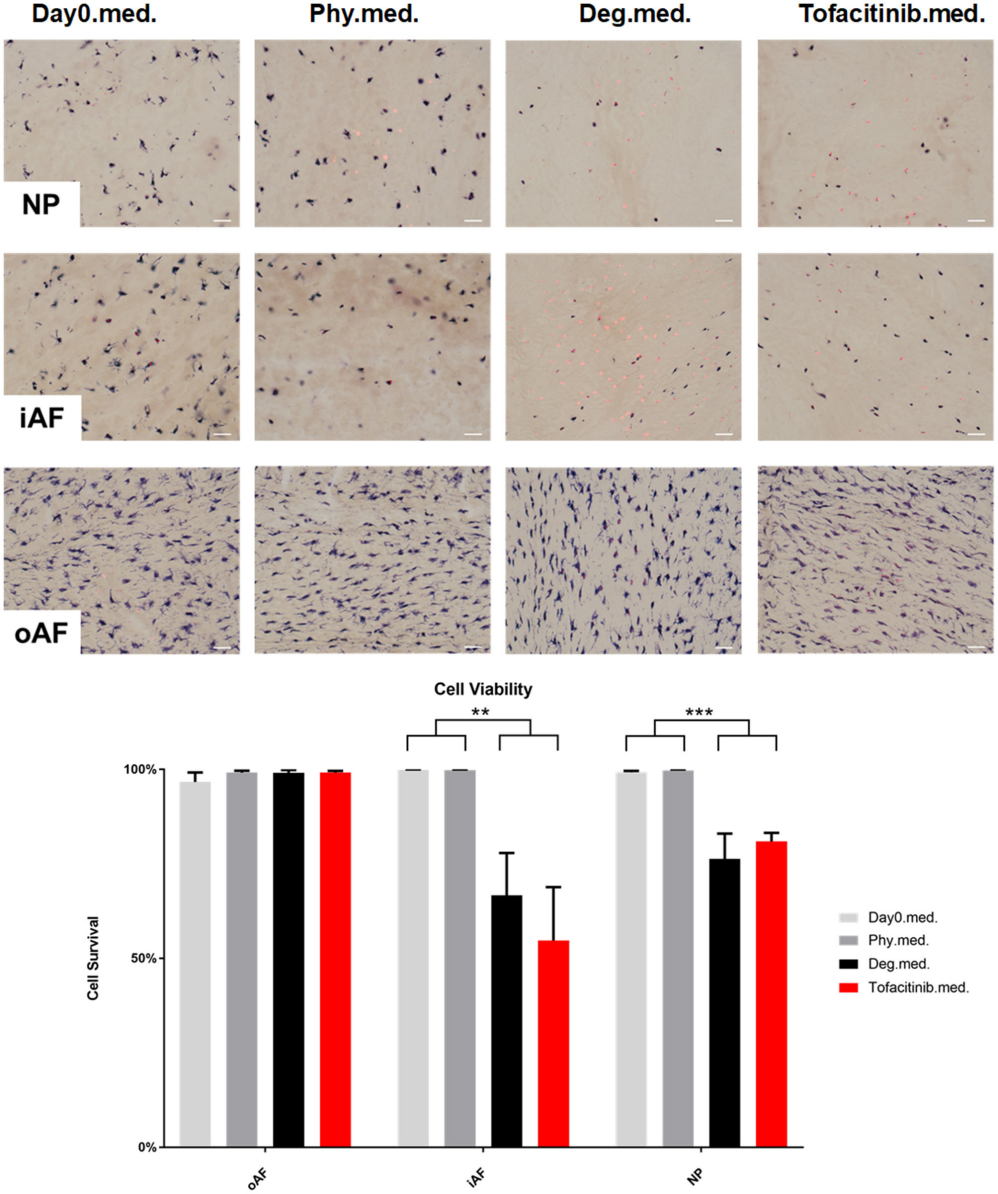
Cell viability of IVD tissue from the Tofacitinib experiments. Representative LDH/ EthD-1 staining image of NP region, inner AF region (iAF) and outer AF (oAF) region from day 0 control samples (Day0.med.), IVDs cultured under physiological condition on day 4 (Phy.med.), IVDs cultured under degenerative condition on day 4 (Deg.med.), and IVDs cultured under degenerative condition and treated with Tofacitinib on day 4 (Etanercept.med.). Scale bar: 50 μm. The percentage of alive cells were counted, as presented in the bar graph. *n* = 8, Means + SEM, **p < 0.01, ***p < 0.001.

Cell viability of disc tissue was assessed after 4 days of culture. Degenerative loading combined with TNF-α injection decreased cell viability in the NP and iAF regions compared to the Phy and Day0 control groups (*p* < 0.01; Figures 13, 14), whereas viability in the oAF tissue was not influenced by Deg treatment after 4 days of culture. Neither of the drugs did affect the cell viability under Deg condition during the observation time (Figures 13, 14).

### Disc Height Change

**Figure 15:**
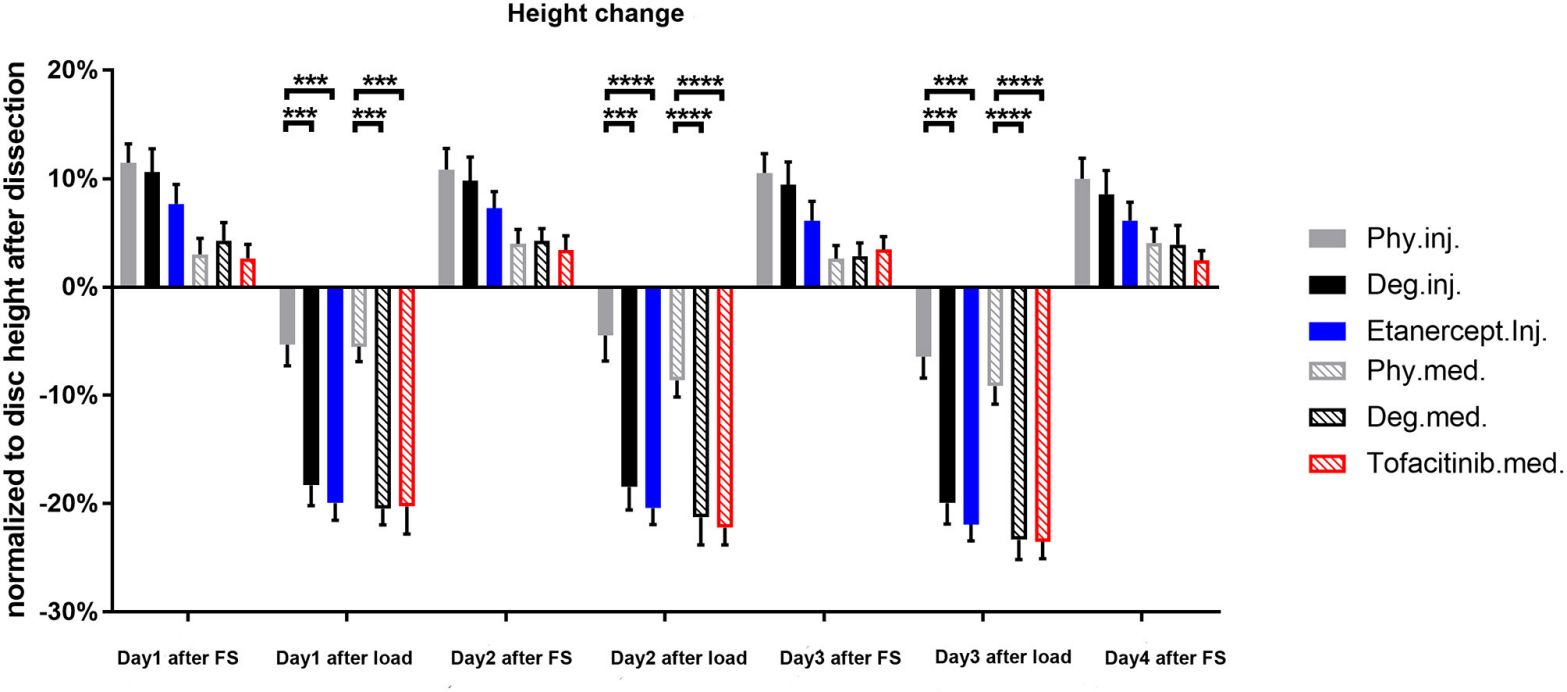
Disc height change normalized to the original dimension after dissection. *n* = 8, means ± SEM, ***p < 0.001, ****p < 0.0001, FS, Free swelling.

Degenerative loading and detrimental culture caused a significantly stronger disc height loss (~20%) compared to the physiological loading protocol and culture (~10%, *p* < 0.001) (Figure 15). During free swelling recovery, all IVDs could recover to the initial disc height before first loading (~105–110%). This diurnal disc height change pattern was observed throughout the entire period of 3 days of repetitive dynamic load. The height loss in the detrimentally loaded groups seemed to increase by time, indicating progressive destruction of tissue. Whereas, the extent of height recovery demonstrated consistency. The drugs did not alter the potential of recovery or the height loss after loading during the observation time.

## Discussion

### Detrimental Loading, Nutrient Deficiency, and TNF-α Injection Simulates Early-Stage Intervertebral Disc Degeneration

Proinflammatory cytokines are considered of crucial importance in the pathogenesis of IDD as they link the inflammatory process to accelerated tissue degeneration and pain. Previous work provided evidence, that the expression of proinflammatory cytokines such as TNF-α, IL1β, IL-6, and IL-8, is associated with the disease activity of IDD in human subjects and animal models (Le Maitre et al., 2005, 2007b; Bachmeier et al., 2007; Hoyland et al., 2008; Freemont, 2009; Freeman et al., 2013; Purmessur et al., 2013; Andrade et al., 2016; Sutovsky et al., 2017). In the present study, degenerative culture conditions combined with TNF-α injection stimulated proinflammatory gene expression (IL-1β, IL-6, and IL-8 in the NP and IL-1β, IL-8, and COX2 in the AF) and upregulated catabolic enzymes (MMP1 and MMP3) with a more profound increase in the NP compared with the AF tissue. These results support the work by Walter et al. and confirm our TNF-α intradiscal injection model, with a higher TNF-α gradient in the NP than in the AF (Walter et al., 2015a; Lang et al., 2018).

Interestingly, degenerative and proinflammatory culture condition also induced gene expression of nerve growth factor (NGF) in the AF tissue, which is related with nociceptive nerve ingrowth and discogenic pain (Freemont et al., 1997; Nakawaki et al., 2019). Previous work has already indicated that, inflammatory cytokines such as IL-1β, TNF-α regulate local intradiscal NGF expression and subsequent ingrowth of small non-myelinated nerve fibers into the IVD (Abe et al., 2007; Miyagi et al., 2012, 2018; Nakawaki et al., 2019). Although our model lacks the opportunity to investigate pain behavioral changes due to IDD driven NGF expression as done by others (i.e., *in vivo* studies in rodents), upregulation of NGF in the AF tissue may be a hint for local intradiscal NGF production in our *ex vivo* system as observed in symptomatic IDD (Lai et al., 2015).

Additionally, degenerative culture conditions combined with TNF-α injection elevated the IL-8 protein release compared to physiological culture conditions, further indicating inflammatory disc condition consistent with our previous work (Lang et al., 2018). Likewise, GAG and NO release were enhanced due to the inflammatory and degenerate culture condition. The collagen type II and proteoglycan expression in the IVD were not reduced by degenerative culture condition compared to the Phy group, as indicated by IHC staining in the current study and Safranin O staining and GAG content quantification from previous study, respectively (Lang et al., 2018). This is due to the high intrinsic content of GAG and collagen type II within the young bovine discs, which did not show a reduction under Deg condition during the culture period. However, the enhanced GAG release into the culture medium indicates the early onset of matrix degradation.

The biomechanical response of IVDs under a degenerative proinflammatory stimulus was assessed via disc height change. Outcome indicates that a short-term application of degenerative loading increased temporary disc height loss of bovine IVDs. However, the disc height recovery capacity after free swelling did not differ between the groups, which is consistent to our previous study (Lang et al., 2018). Degenerative loading combined with TNF-α injection significantly decreased cell viability in the NP and inner AF tissue compared to the physiological and Day0 control groups.

In summary, the combination of detrimental dynamic loading, nutrient deficiency, and intradiscal TNF-α injection is able to simulate the proinflammatory and degenerative condition operative in IDD, highlighting the potential of our whole IVD organ culture model to efficiently screen and explore novel anti-inflammatory agents or regenerative therapies.

### Etanercept and Tofacitinib Reduce Proinflammatory Activity in IDD

As far as we know, this is the first study evaluating anti-inflammatory effects of Etanercept and Tofacitinib within a proinflammatory and degenerative microenvironment of a whole organ IVD culture model. Both, Etanercept and Tofacitinib partially prevented the upregulation of proinflammatory cytokines in IVDs cultured under IDD-like conditions which supports outcome of previous *in vitro* studies on TNF-α inhibition (Likhitpanichkul et al., 2015; Walter et al., 2015b; Evashwick-Rogler et al., 2018). Moreover, our data confirms the concept of recent work investigating a potential involvement of the IL-6/JAK/STAT3 pathway in IDD (Suzuki et al., 2017). Pharmacological inhibition of JAK3 activity with CP690550 significantly suppressed the IL-6-mediated gene expression in human AF cells. Increased expression levels of IL-6 are associated with active discopathy (Kang et al., 1995; Watts et al., 1996; Pedersen et al., 2015). Milici et al. performed a rodent *in vivo* study to evaluate whether selective JAK3 inhibition can preserve cartilage in RA. Outcome revealed a dose dependent reduction of clinical and histological signs of joint inflammation following JAK-inhibition (Milici et al., 2008).

On protein level, Etanercept and Tofacitinib did not alter IL-8 protein release. Etanercept partially diminished the elevated release of proinflammatory mediator NO compared to IVDs cultured within a degenerative and proinflammatory microenvironment, while Tofacitinib did not. In summary, these results indicate a superior protective anti-inflammatory effect of Etanercept in the tested conditions compared with Tofacitinib. Within the IVD tissue, a region dependent anti-inflammatory effect of both drugs was observed, where Etanercept reduced the percentage of IL-1β positively stained cells in the oAF region, while Tofacitinib reduced the percentage of IL-1β and IL-8 positively stained cells in the iAF region.

### Etanercept Shows a “Pain” Alleviation Effect

Intradiscal administration of Etanercept has shown to relieve pain at 4-weeks after injection in discogenic low back pain patients (Sainoh et al., 2016). In the current study, Etanercept reduced the expression of NGF in the AF tissue. Previous research by Kivitz et al. (2013) and Katz et al. (2011) investigated whether Tanezumab, a monoclonal antibody that specifically inhibits NGF, relieves chronic pain in a randomized controlled trial against naproxen and placebo. The authors concluded that Tanezumab provides greater improvement in pain, function, and global scores vs. placebo and naproxen in patients with chronic low back pain. These results indicate that Etanercept may alleviate discogenic pain by inhibition of NGF expression.

### Etanercept and Tofacitinib Partially Diminish Matrix Catabolism in IDD

Etanercept and Tofacitinib prevented the upregulation of MMP1 and MMP3 catabolic gene expression in the NP tissue. In addition, both drugs reduced the GAG loss to the level under physiological culture condition. These results indicate that both drugs could prevent matrix degradation and slow down the catabolic pathology in disc degeneration. They are also consistent with previous research, where Tofacitinib was shown to reduce degenerative effects of proinflammatory cytokines in rat AF cells (Suzuki et al., 2017). IVD degeneration starts with GAG loss and fibrosis in the tissue. Therefore, these drugs which showed an inhibition effect on GAG loss under the degenerative condition are of important potential for clinical translation, to slow down the IVD degeneration process after traumatic injury.

Both biologicals tested in the present study are FDA approved drugs for the treatment of chronic inflammatory diseases such as RA, Psoriasis, and ulcerative colitis. These drugs have been implemented in daily clinical use with limited clinical side effects (Genevay et al., 2009; Fleischmann et al., 2012). Furthermore, Etanercept was proved successful in alleviating symptoms in patients with disabling discogenic or radicular LBP (Genevay et al., 2004, 2012; Cohen et al., 2009; Okoro et al., 2010; Ohtori et al., 2012; Freeman et al., 2013; Sainoh et al., 2016).

### Strengths and Limitations

In contrast to previous models, the present organ culture system comprises a 3D IVD microenvironment combined with dynamic loading, which is more relevant than cell culture experiments (Ponnappan et al., 2011; Purmessur et al., 2013; Walter et al., 2015a; Krupkova et al., 2016; Teixeira et al., 2016). *In vitro* studies have several limitations when assessing inflammation and IVD homeostasis as the monolayer culture itself induces a dedifferentiation effect on IVD cell phenotype (Kluba et al., 2005). IVDs can be maintained viable under dynamic load in a bioreactor for several weeks while still having intact endplates (Illien-Junger et al., 2010; Li et al., 2015, 2016a, 2017). Here, we offer a relevant and easily modifiable whole organ culture system providing a straightforward and cost-efficient approach to rapidly screen a variety of therapies for IVD regeneration. Furthermore, the present organ culture system reduces the need for unnecessary animal studies and thus perfectly represents the 3R principles “reduce, replace, refine” while still delivering relevant answers on research questions. Probably, the observed changes on gene signaling are “early signs” which may cause alteration on protein expression when performing longer experiments. Hence, the limited effect of the drugs might be due to the short observation time. The current study was designed as a first screening step to investigate if Etanercept and Tofacitinib are able to inhibit or slow down this inflammation at the beginning of the signaling progression, which is the primary requisite for anti-inflammatory therapeutics that show an immediate response. Further analysis of another longer time point is warranted in future studies to evaluate the long-term effect of the drugs. In our experiments, neither Etanercept nor Tofacitinib were capable of maintaining initial cell viability during degenerative culture. Though targeting different signaling molecules, both drugs could partially reduce the detrimental effects. However, combined anti-inflammatory and anabolic treatment may be required to constrain accelerated IVD degeneration while relieving back pain and promoting disc regeneration.

Changes on gene expression level are the first alterations in the onset of degeneration which are later followed by protein and structural changes. The distinctive distribution of cytokines among different disc tissue regions in symptomatic IDD might offer a basis for tissue specific application of novel treatments, which needs further intensified investigation.

In addition, systemic reaction and interaction cannot be investigated in the present organ culture system. Certainly, this system fails to reproduce complex immunologic interactions. However, intradiscal injection of Etanercept in patients already demonstrated beneficial effects which were partially reproducible in our bioreactor system (Tellegen et al., 2018).

## Conclusions

The combination of detrimental dynamic loading, nutrient deficiency and intradiscal TNF-α injection could simulate the proinflammatory and degenerative condition operative in active discopathy, highlighting the potential of our whole IVD organ culture model to efficiently screen and explore novel anti-inflammatory agents or regenerative therapies. In addition, Etanercept and Tofacitinib revealed their capability to slow down the degenerative cascade and neutralize the proinflammatory microenvironment in our early onset organ culture model. Combined anti-inflammatory and anabolic treatment may be required to constrain accelerated IVD degeneration while relieving back pain and promoting disc regeneration.

## Data Availability Statement

Datasets are available on request. The raw data supporting the conclusions of this article will be made available by the authors, without undue reservation, to any qualified researcher.

## Author Contributions

ZL: substantial contributions to study design, acquisition, analysis, interpretation of data, drafting the paper, revising it critically, and final approval. YG and FH: substantial contributions to acquisition of data, analysis, interpretation of data, revising the article critically, and final approval. DK, NS, KI, EK, RR, and MA: substantial contributions to study design, revising the article critically, and final approval. SG: substantial contributions to study design, interpretation of data, revising the article critically, and final approval. GL: substantial contributions to study design, interpretation of data, drafting the article, revising it critically, final approval, and takes responsibility for the integrity of the work as a whole, from inception to finished article.

## Conflict of Interest

The authors declare that the research was conducted in the absence of any commercial or financial relationships that could be construed as a potential conflict of interest.

## Supplementary Material

Supplementary Figure 1 Gene expression levels of NP tissue in Tofacitinib dose response experiment. Gene expression levels of NP tissue after 4 days of culture under Deg culture condition, with 250 ng/mL or 2.5 μg/mL Tofacitinib application into the medium, normalized to Phy group. *n* = 2. NP, Nucleus pulposus; IL, Interleukin; COX-2, Cyclooxygenase-2.
