# Supplementary material for: Preclinical ex-vivo Testing of Anti-inflammatory Drugs in a Bovine Intervertebral Degenerative Disc Model
Source: Front Bioeng Biotechnol. 2020 Jun 10;8:583. doi: 10.3389/fbioe.2020.00583 (PMC7298127; doi:10.3389/fbioe.2020.00583)
Supplement: Supplementary file 1 [file Table_1.docx]

**Supplementary Table**

**Table 1. Custom designed Primers and Probes**

| Gene |  | |
| --- | --- | --- |
| bMMP1  (Matrix Metalloprotease 1) | Forward primer seq  reverse primer seq  probe seq  5' modification  3' modification | 5'-TTC AGC TTT CTC AGG ACG ACA TT-3'  5'-CGA CTG GCT GAG TGG GAT TT-3'  5'-TCC AGG CCA TCT ACG GAC CTT CCC-3'  FAM  TAMRA |
| bMMP3  (Matrix Metalloprotease 3) | Forward primer seq  reverse primer seq  probe seq  5' modification  3' modification | 5'-GGC TGC AAG GGA CAA GGA A-3'  5'-CAA ACT GTT TCG TAT CCT TTG CAA-3'  5'-CAC CAT GGA GCT TGT TCA GCA ATA TCT AGA AAA C-3'  FAM  TAMRA |
| bMMP13  (Matrix Metalloprotease 13) | Forward primer seq  reverse primer seq  probe seq  5' modification  3' modification | 5'-CCA TCT ACA CCT ACA CTG GCA AAA G-3'  5'-GTC TGG CGT TTT GGG ATG TT-3'  5'-TCT CTC TAT GGT CCA GGA GAT GAA GAC CCC-3'  FAM  TAMRA |
| bIL-1β  (Interleukin-1β) | Forward primer seq  reverse primer seq  probe seq  5' modification  3' modification | 5'-TTA CTA CAG TGA CGA GAA TGA GCT GTT-3'  5'-GGT CCA GGT GTT GGA TGC A-3'  5'-CTC TTC ATC TGT TTA GGG TCA TCA GCC TCA A-3'  FAM  TAMRA |
| bIL-6  (Interleukin-6) | Forward primer seq  reverse primer seq  probe seq  5' modification  3' modification | 5'-TTC CAA AAA TGG AGG AAA AGG A-3'  5'-TCC AGA AGA CCA GCA GTG GTT-3'  5'-CTT CCA ATC TGG GTT CAA TCA GGC GATT-3'  FAM  TAMRA |
