# Supplementary figures and images for: Preclinical ex-vivo Testing of Anti-inflammatory Drugs in a Bovine Intervertebral Degenerative Disc Model
Source: Front Bioeng Biotechnol. 2020 Jun 10;8:583. doi: 10.3389/fbioe.2020.00583 (PMC7298127; doi:10.3389/fbioe.2020.00583)

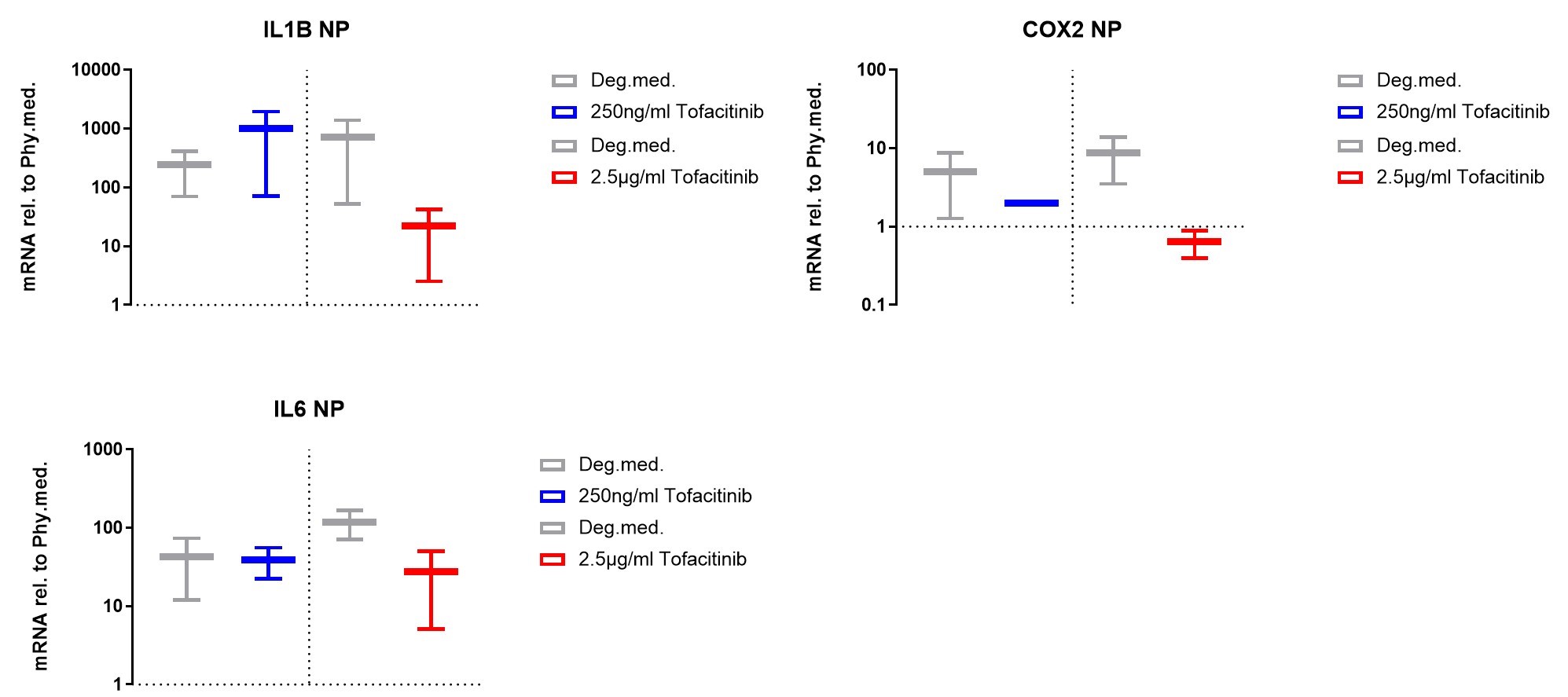

Supplement: Supplementary Figure 1 — Gene expression levels of NP tissue in Tofacitinib dose response experiment. Gene expression levels of NP tissue after 4 days of culture under Deg culture condition, with 250 ng/mL or 2.5 μg/mL Tofacitinib application into the medium, normalized to Phy group. n = 2. NP, Nucleus pulposus; IL, Interleukin; COX-2, Cyclooxygenase-2. [file Image_1.JPEG]
